# From task-general towards task-specific cognitive operations in a few minutes? Working memory performance as an adaptive process

**DOI:** 10.1177/17470218241278272

**Published:** 2024-09-18

**Authors:** Jussi Jylkkä, Zachary Stickley, Daniel Fellman, Otto Waris, Liisa Ritakallio, Todd D Little, Juha Salmi, Matti Laine

**Affiliations:** 1 Department of Psychology, Åbo Akademi University, Abo, Finland; 2 College of Education, Texas Tech University, Lubbock, TX, USA; 3 Department of Clinical Neuroscience, Karolinska Institutet, Stockholm, Sweden; 4 Department of Applied Educational Science, Umeå University, Umeå, Sweden; 5 Department of Child Psychiatry, University of Turku and Turku University Hospital, Turku, Finland; 6 INVEST Research Flagship Center, University of Turku, Turku, Finland; 7 Optentia Research Focus Area, North-West University, Vanderbijlpark, South Africa; 8 Department of Neuroscience and Biomedical Engineering, Aalto University, Finland

**Keywords:** Working memory, skill learning, longitudinal structural equation modelling, latent structure, cognitive architecture, cognitive task dynamics

## Abstract

Measurement of cognitive functions is typically based on the implicit assumption that the mental architecture underlying cognitive task performance is constant throughout the task. In contrast, skill learning theory implies that cognitively demanding task performance is an adaptive process that progresses from initial heavy engagement of effortful and task-general metacognitive and executive control processes towards more automatic and task-specific performance. However, this hypothesis is rarely applied to the short time spans of traditional cognitive tasks such as working memory (WM) tasks. We utilised longitudinal structural equation models on two well-powered data sets to test the hypothesis that the initial stages of WM task performances load heavily on a task-general g-factor and then start to diverge towards factors specific to task structure. In line with the hypothesis, data from the first experiment (*N* = 296) were successfully fitted in a model with task-initial unity of the WM paradigm-specific latent factors, after which their intercorrelations started to diverge. The second experiment (*N* = 201) replicated this pattern except for one paradigm-specific latent factor. These preliminary results suggest that the processes underlying WM task performance tend to progress rapidly from more task-general towards task-specific, in line with the cognitive skill learning framework. Such task-internal dynamics has important implications for the measurement of complex cognitive functions.

## Introduction

Cognition is an adaptive process. Novel tasks call for adaptive behaviour that is thought to rely on successful executive control (Miller & Cohen, 2001; Miyake et al., 2000; Norman & Shallice, 1986). Although adaptivity and learning are considered to be cornerstones of human cognitive ability (Anderson, 2014; Chein & Schneider, 2012), the outcome measures of complex cognitive task performances typically sum up the whole task period into single scores. This entails a static view of the mental architecture underlying task performance. Anderson (2014) calls this the *componential computational theory of mind*, which implies that the mind consists of dissociable functions that can be measured with cognitive tasks. For example, the Stroop task is thought to tap on peoples’ inhibitory capacity, whereas the *n*-back task reflects their working memory (WM) updating capacity. Considering cognition as an adaptive process is a radical departure from this approach—it entails that performing a cognitive task alters what is being measured, because the cognitive system adapts to the testing context. Thus, what is measured by a task is partly created by the measurement situation, rather than being “out there” on the latent level, waiting to be measured. This way of thinking motivates examining cognitive task performance as a dynamic process that evolves over time.

The cognitive skill learning approach (Ackerman, 1988; Chein & Schneider, 2012; Schneider & Chein, 2003; Schneider & Shiffrin, 1977; Shiffrin & Schneider, 1977) espouses this general view by focusing on the adaptivity of cognition. It is based on a long research tradition on slow and effortful versus fast and automatic cognitive systems. The skill learning approach is often applied to explain learning in perceptual-motor tasks or tasks that require problem solving. In this study, we applied it to complex cognitive task performance, namely WM tasks. Besides involving perceptual-motor processes, the cognitive demands and unfamiliarity of WM tasks call for executive resources needed for strategy generation and implementation (Waris, Fellman, et al., 2021; Waris, Jylkkä, et al., 2021). In this way, they can be considered as analogical to any cognitively demanding novel task that requires skill to be performed efficiently. The cognitive skill learning approach entails that processing of novel tasks initially relies heavily on task-general metacognitive and control processes that through repeated practice are gradually replaced by more task-specific, automatised processes (Taatgen, 2013). Metacognitive processes include the generation of a suitable strategy for the task, whereas the controlled execution phase, engaging the executive system, involves its effortful and controlled implementation. In turn, automatisation proceeds gradually with task exposure and frees the effortful processes to other tasks (Chein & Schneider, 2012).

Applying the cognitive skill learning approach to the psychometric context implies that what is being measured can change as the participant develops a skill to perform the task. Consider a complex WM task like the *n*-back, where the participant sees stimuli that are constantly changing. During each new stimulus, the participant needs to decide whether the stimulus is the same as the one that was presented *n* trials ago. For example, in a 2-back task with the series 2-5-1-**5**-4-3-**4**, the subject should press the response button corresponding to “same” during the trials that are bolded above (Kirchner, 1958). The participant assumedly enters the metacognitive phase already upon receiving task instructions (wondering what the task will be like, trying to remember the stimulus-response mappings and task instructions, starting to plan a strategy, etc.). The memorised task instructions and self-generated strategies are effortfully implemented, monitored, and adjusted in the beginning of a task, but with continued performance, processing is gradually automatised. Thus, following the skill learning view (Chein & Schneider, 2012), we hypothesise that a demanding cognitive task relies on different processes depending on the stage of learning. Here, we studied whether signs of such changes could be detected already during the brief time spans (minutes) that commonly employed WM tasks usually take.

The assumption that the later stages of task performance serve to consolidate task-specific skills receives support from the cognitive training literature, but there the time scales span from hours to weeks, rather than minutes. Prolonged training on a cognitive task typically does not yield transfer to other types of tasks, but rather enhances performance only on tasks that have close structural resemblance to the trained task (Au et al., 2015; Karbach & Verhaeghen, 2014; Kassai et al., 2019; Melby-Lervåg et al., 2016; Melby-Lervåg & Hulme, 2013; Sala & Gobet, 2017; Schwaighofer et al., 2015; Weicker et al., 2016). For example, *n*-back training with digit stimuli can clearly enhance performance on an untrained *n*-back task with letters, but not on structurally dissimilar WM updating tasks such as a running memory task (Gathercole et al., 2019; Soveri et al., 2017). This lack of transfer could be explained by considering cognition as an adaptive process, where performance on a repeated task starts to rely more and more on task-specific subroutines instead of task-general processes (Fellman et al., 2020). Also, the functional brain imaging studies of WM training, showing modulations of activity in large-scale brain networks associated with cognitive and perceptual-motor systems, are consistent with the cognitive skill learning framework on this longer time scale. Activity in the dorsolateral prefrontal cortex as well as parietal and sensory areas, initially responding to high cognitive load, attenuates over the training period, whereas activity of the striatum that is involved in executing learned sequences increases (Salmi et al., 2018).

This study differs crucially from the abovementioned WM training studies by examining the evolvement of task paradigm-based latent structure of commonly used WM tasks within the short time span of a single testing session that took ca. 5 to 14 minutes per task (“paradigm” refers here to the type of task, e.g., variants of the *n*-back task represent the *n*-back paradigm). Two lines of previous evidence motivated us to apply this approach to such short time spans. First, there is neuroimaging evidence indicating that the brain systems underlying WM begin to adapt already during a single testing session (Badre et al., 2010). Another line of empirical evidence for the dynamic nature of the cognitive processes underlying within-session WM performance comes from self-reported strategies that are also reliably associated with objective WM performance (Fellman et al., 2020; Forsberg et al., 2020; Laine et al., 2018). Of particular relevance here is a microgenetic study (Waris, Jylkkä et al., 2021) that analysed the block-by-block evolvement of spontaneous strategy use within a single *n*-back test session. About half of the participants reported using a self-generated strategy already during the very first task block, and changes in selected strategy were most common during the initial task blocks, after which strategy use became more stable. The generality of this within-task strategy development pattern was corroborated by three similar block-by-block strategy analyses that employed quite different episodic and prospective memory tasks (Laine, Fellman, et al., 2024; Laine, Jylkkä, et al., 2024; Waris, Fellman et al., 2021). The findings of these microgenetic studies fit well to the cognitive skill learning view, according to which strategy generation and controlled execution (including monitoring and strategy adaptation), which represent task-general executive resources, are most prominently in use during the initial stages of cognitively demanding task performance.

In this study, pretest data from two previously published WM training experiments were employed (Fellman et al., 2020; Ritakallio et al., 2022). We analysed these two data sets to enable replication. At pretest, participants saw the WM tasks for the first time. The WM task paradigms were *n*-back, running memory, simple span, and selective updating (SU), with partly different paradigm and task constellations in the two experiments. Each WM paradigm included task variants that employed different stimuli. To create a longitudinal repeated measures data matrix, we divided each 5- to 14-minute task period into four approximately equally long phases and calculated each participant’s performance scores for each phase.

We analysed these data with longitudinal structural equation modelling (LSEM), a latent variable modelling approach with multiple observed indicators. Using LSEM allowed us to separate measurement error from true individual differences linked to variability and/or change over time. Our measurement structure was as follows: the latent variables were the paradigm-specific factors (*n*-back, running memory, simple span, SU), and their observed indicators were the participants’ performance scores on the task variants of each WM paradigm at each time point. This data structure was subjected to test our hypothesis that these latent WM factors load heavily enough on general executive resources to form unity at the initial phase or phases but not later. Prior to hypothesis testing, we had to first establish measurement invariance, meaning that the expected changes happened in the paradigm-specific latent factors and not in the measurement properties of their observed indicators (the WM tasks). After that, hypothesis testing ensued. This was done step-by-step by introducing hypothesis-guided constraints to the latent factors’ covariance structure and observing the subsequent model fit, finally arriving at a model that was as parsimonious as possible without losing fit to the data. As our hypothesis predicted a change from relative unity towards diversity in the covariance structure, hypothesis testing proceeded by constraining latent factor intercorrelations to 1.00 (reflecting the g-factor of task-general executive control) whenever that was possible without significantly worsening model fit and examined to what extent the resulting constrained covariance structure followed the hypothesised pattern of change. Due to the variability of the WM tasks and the lack of previous research, we did not attempt to predict at which time point the latent factor intercorrelations would start to decline. All in all, the goal of this LSEM approach was to attain the most simple and parsimonious fit to the covariance structure of the data, allowing for evaluation of the resulting model in light of our hypothesis. This methodology has previously been used in multiple group comparisons and intervention studies (for a review, see Little et al., 2022); however, in our case, the longitudinal performance data consisted of a single group of participants who took a battery of WM tasks.

## Experiment 1

In this experiment, we tested our hypothesis that the latent factor intercorrelations evolve from relative unity towards paradigm-specific diversity with data from an Internet-based WM training study. To examine the underlying cognitive architecture of WM processing when the tasks are novel, we focused solely on pretest performances. The tasks represented commonly used WM paradigms, each lasting on average 5 to 14 minutes. The data and the scripts used in the analyses are available at https://osf.io/gvqhu/.

### Participants and procedure

The current data stem from the pretest of an online randomised controlled trial that examined WM training and its mechanisms (Fellman et al., 2020). The trial was preregistered (see https://aspredicted.org/r7qs9.pdf) but because the data were collected for other purposes, the preregistration did not include the present hypothesis. The study was approved by the Institutional Review Board of the Departments of Psychology and Logopedics, Åbo Akademi University, and it was conducted in accordance with the Helsinki Declaration. All participants gave their written informed consent. In this study, 419 participants were recruited through Prolific Academic (https://www.prolific.co/). Of these, 296 participants were included in this study after excluding those that did not meet the inclusion criteria (*n* = 117), and those that reported cheating during task performance (*n* = 6). The inclusion criteria were English as native tongue, no current psychiatric or neurological illnesses that affected daily life, no current use of central nervous system (CNS) medication, no current psychotropic drug use (except tobacco, alcohol, and cannabis), and no intoxication at the time of testing. The participants were 18- to 50-year-olds with a mean age of 34.08 years (*SD* = 8.52), and 62.50% (*n* = 185) were females. The mean length of education in the sample was 15.80 years (*SD* = 3.49).

### Materials

The WM tasks included in this study encompassed four *n*-back tasks (separate tasks with digits, letters, colours, and boxes as stimuli), four simple span tasks (forward simple spans with digits and boxes, backward simple spans with digits and boxes), and two running memory tasks (digits and boxes). All data were from the pretest of the original experiment when the participants saw these tasks for the first time (Fellman et al., 2020).

#### *n*-Back

This task calls for continuous creation and updating of arbitrary bindings between items and their positions in a stimulus sequence. This requires executive control as it is prone to interference (Szmalec et al., 2011). We employed four adaptive single *n*-back tasks with different stimuli, namely digits (1–9), letters (A–I), colours (blue, yellow, red, green, purple, black, pink, orange, and grey) and locations (boxes presented in a 3 × 3 matrix) as stimuli. In each *n*-back task variant, the stimuli were presented one at a time on a computer screen. The participants were to respond “yes” or “no” to each item with a keyboard press, indicating whether the current item corresponded to the item presented *n* items back in the stimulus sequence. Each *n*-back variant included 12 task blocks, with 20 + *n* trials in each block. Out of the 20 trials in a block, six were targets and 14 were nontargets. To increase task demands, four nontargets were lures (i.e., identical to the target items but presented *n* ± 1 back). Each stimulus was displayed for 1500 ms, and the interstimulus interval was 450 ms. The *n*-back tasks were adaptive so individual success rate determined the level of task difficulty. Each *n*-back task started always with a 1-back block, and the level of *n* could vary between 1 and 12. If the participant responded correctly on 18 to 20 trials in a block, the programme increased *n* by one. The level of *n* remained unchanged if the participant recalled 15 to 17 trials correctly, whereas five or more incorrectly recalled trials resulted in a decrease of *n* by one. Each *n*-back task took about 14 min. No practice trials were included as the task started at the easiest 1-back level.

#### Span

A classical method in the evaluation of WM capacity, span tasks call for short-term maintenance of memory contents, either with or without the need to manipulate their order. There were four simple span tasks: two forward span and two backward span tasks. One forward span task employed digits as stimuli (forward simple span with digits; FSD), and the other one visuospatial locations (forward simple span with boxes; FSB). In both variants, there was one trial for each list length, and the length of the randomised lists ranged from 4 to 10 in the FSD and 3 to 9 in the FSB. Also the backward simple span tasks had one variant with digits (backward simple span with digits; BSD) and the other one with locations (backward simple span with boxes; BSB). For both Backward span variants, the list lengths varied from 3 to 9, and the sequences were presented in a randomised order. Similarly to the Forward simple span tasks, the participants were shown one sequence per list length. For both the forward and backward simple span task, the item exposure time was 1000 ms and the interstimulus interval 500 ms. Each span task took ca. 5 min. Depending on the participant’s performance, there were three to seven practice sequences. The final practice sequence was nine items long, whereas the other ones had four to five items. Recalling correctly two consecutive short sequences, the participant proceeded to the final longer sequence. After that, the actual task commenced irrespective of the correctness of the recall of the longer sequence.

#### Running memory

In this task, the participants are to recall a given number of last items from a sequence that is suddenly aborted. It is typically assumed that running memory performance calls for updating the list of most recently presented items in WM. We employed two task variants, a running memory task with digits (RMD) and a running memory task with boxes (RMB). For both task variants, stimulus presentation time was 1000 ms, with an interstimulus interval of 500 ms. In RMD, the participants saw digit sequences with unpredictable length on the computer screen, after which they were to recall the last four items in correct order. The RMB was identical to the RMD, except that the stimuli were spatial locations in a 3 × 3 matrix. For both task variants, the participants completed eight trials in a randomised order. The item sequences varied from 4 to 11 items, with one trial per each list length. The running memory tasks took ca. 6 min each. Depending on participant’s performance, there were two to six practice sequences. Correct recall of two consecutive sequences led to the actual task.

### Dependent variables

To examine the changes in latent factor intercorrelations over time, we split the task blocks into four phases, roughly matched in length. This was done to attain a balance between having enough observations to enable a sufficiently reliable analysis versus having a short time span in case the intercorrelations would change relatively quickly. The *n*-back tasks, i.e., *n*-back task with digits (NBD), *n*-back task with letters (NBL), *n*-back task with colours (NBC), and *n*-back task with boxes (NBB), were divided into blocks of three (3/3/3/3), whereas the other tasks (i.e., the running memory tasks RMD and RMB and the span tasks FSD, FSB, BSD, and BSB) were divided into blocks of one and two (1/2/2/2). Thus, where an even split was impossible, the initial phase was kept shorter. This was motivated by the assumption that most changes in cognitive skill learning happen at the initial stages of task performance (Chein & Schneider, 2012). The dependent variable for the *n*-back tasks was the average *n*-back level achieved in the respective task phase. For the running memory tasks, the dependent variable was calculated by counting the correctly recalled items in correct position in a sequence, and then averaging the scores over sequences within the respective task phases. For the span tasks, the dependent variable was the proportion of correctly recalled items in correct order, that is, the number of correctly recalled items/total number of items.

### Analytical approach

LSEM over the four task phases was conducted by employing the *lavaan* package version 0.6-9 (Rosseel, 2012) in the R environment version 4.1.1 (R Core Team, 2021) using Rstudio version 2021.9.0.351 integrated development environment (R Studio Team, 2021). Goodness of fit was evaluated with the model’s χ^2^, the comparative fit index (CFI), the Tucker–Lewis index (TLI), the root mean square error of approximation (RMSEA), and the standardised root mean squared (SRMR). Acceptable model fit was determined by holistically evaluating the model fit indices, and acceptable values were set using the Little (2013) guidelines of CFI and TLI ⩾0.900, RMSEA and SRMR ⩽0.080. Item reliability was evaluated using McDonald’s ω coefficient (McDonald, 1999). McDonald’s ω coefficient can be interpreted using the same heuristics as Cronbach’s α coefficient and reports the proportion of variance in common among the items as part of the total observed variance. Measurement invariance was evaluated using the change in CFI of <0.01 (Cheung & Rensvold, 2002; Little, 2013), and tests of latent parameter differences were evaluated using the χ^2^ difference test at an alpha level of .05 to determine significance.

The analysis proceeded in two stages, first determining measurement invariance, and then examining how correlations between the latent factors change over time. Invariance testing establishes whether the items being used are psychometrically equivalent at each time point, allowing us to conclude that any changes in the constructs are changes in the constructs themselves and not changes in the measures being used. It is noteworthy that although the cognitive skill learning hypothesis implies a change in latent factor structure over time from relative unity towards task-specific structure, testing this hypothesis requires measurement invariance, which is a prerequisite for examining changes in factor intercorrelations (Little, 2013).

To evaluate the change in factor intercorrelations over time, we first examined whether it is possible to fit the data by constraining all the paradigm-specific latent factor intercorrelations to unity (*r* = 1.00) at each time point in sequence, starting with the initial task phase. We expected that it is possible to constrain the factors to unity at the initial phase or phases but not at later ones (our hypothesis does not state when exactly the factors diverge, only that they exhibit relative unity in the beginning). We then examined in more detail what is the most parsimonious model by constraining the correlations between each pair of the paradigm-specific latent factors separately. This was done to examine which of the latent factors form relative unity, if any, and at what time points. In this stepwise process, the initial unconstrained model was used as a baseline for identifying correlations that appeared to be similar in magnitude. First, correlations that appeared to be similar in magnitude within time (irrespective of the strength of that correlation) were constrained to equality if this was possible without worsening model fit. This constraint was not done to test any hypothesis, but instead to produce the most parsimonious model and to minimise the number of parameters to be estimated, increasing statistical power. Second, those within-time intercorrelations that appeared to be near 1.00 were constrained to 1.00 if this could be done without significantly worsening model fit. This was done to examine whether any given two factors are unitary within a time point. As noted above, our hypothesis did not state at which specific time point the factor intercorrelations could not anymore be constrained to one, only that this could be done in the beginning and not towards the end. Third, once these within-time correlations were constrained, between-time correlations that appeared to have similar magnitudes (irrespective of the strength of that correlation) were constrained to equality if this was possible without worsening model fit. Similarly to the within-time correlations, this was not done to test any hypothesis but to yield the most parsimonious model for the data and to see between which time points the correlations could be considered as identical versus different. All the changes were evaluated using a χ^2^ difference test, and the final constrained model was the one in which the constraints included did not produce a significantly worse fitting model compared with the unconstrained model, and any additional constraints would produce a significantly worse fitting model. Thus, the final constrained model can be considered as the most parsimonious fit to the data. That model could then be evaluated against our hypothesis that predicts a shift from relative unity towards diversity in the latent factor intercorrelations.

### Results

The data were screened to identify univariate outliers (i.e., scores deviating >3.5 *SD*s from the sample mean on each task); task performances exceeding this threshold were treated as missing. Moreover, performances from participants who reported being colour-blind were treated as missing in the colour tasks. The data set consisted of 296 individuals observed across four time points during each WM task and missingness across all time points ranged from zero to six observations (2%) for any one variable. Our model was estimated using full information maximum likelihood (FIML) to estimate parameters that accounted for the missing observations. As our models utilised all variables in the data set, there were no auxiliary variables to specify. Descriptive statistics and pairwise correlations between tasks are summarised in the Online Supplementary Materials, Appendix A.

The first analytical aim was to establish measurement invariance. Our initial confirmatory factor analysis (CFA) configural model for Experiment 1 demonstrated good fit based on our established criteria with a χ^2^ of 720.448 (*df* = 618), *p*-value of .003, CFI of 0.980, TLI of 0.975, SRMR of 0.046, and RMSEA of 0.024 with a 90% confidence interval of [0.015, 0.031]. For this measurement model, reliability for the *n*-back over the four time points was 0.48, 0.79, 0.86, and 0.88. The reliability for *span* over the four time points was 0.31, 0.53, 0.60, and 0.59. Finally, the reliability for *running memory* over the four time points was 0.23, 0.23, 0.32, and 0.31. Although some of these reliability estimates are low, the loadings for all constructs were significant, allowing comparisons of the factor structure in the disattenuated latent space (Little et al., 1999). This model was evaluated for weak invariance by constraining the item loadings to be equal across time, and then strong invariance by constraining the item loadings and intercepts to be equal across time. Both tests of invariance passed, with a resulting strong model that demonstrated good model fit with a χ^2^ of 778.985 (*df* = 656), *p*-value of .001, CFI of 0.976, TLI of 0.972, SRMR of 0.050, and RMSEA of 0.025 with a 90% confidence interval of [0.017, 0.032]. Fit indices for all levels of invariance can be found in Table 1. Thus, measurement invariance, the prerequisite for examining changes in factor intercorrelations (Little, 2013), was confirmed.

**Table 1.** Experiment 1: Measurement invariance and nested-model comparisons.

	χ^2^	*df*	*p*	CFI	TLI	RMSEA	SRMR	ΔCFI	Δχ^2^	Δ*df*	*p*	Pass?
Configural	720.448	618	.003	0.98	0.975	0.024	0.046	–	–	–	–	Yes
Weak	743.808	635	.002	0.979	0.974	0.024	0.049	0.001	23.359	17	.138	Yes
Strong	778.985	656	.001	0.976	0.972	0.025	0.05	0.003	35.177	21	.027	Yes
Lt. Correlation	787.689	667	.001	0.977	0.973	0.025	0.05	–	8.704	11	.649	Yes
Lt. Variance	785.938	662	.001	0.976	0.972	0.025	0.051	–	6.953	6	.325	Yes
Omnibus	795.043	673	.001	0.976	0.973	0.025	0.052	–	16.058	17	.52	Yes

Measurement invariance was evaluated using the ΔCFI with 0.01 as the threshold for passing. Latent parameters were tested using nested model χ^2^ difference tests with an alpha of .05. “Configural,” “Weak,” and “Strong” refer to the models testing the respective types of invariances; “Lt. Correlation” refers to the model where the factor correlations have been constrained to 1.00 or equality, when that was possible without reducing model fit; “Lt. Variance” refers to the model where the latent score variances were constrained to equality, when that was possible without reducing model fit and “Omnibus” refers to the model where both the correlations and variances have been constrained.

To evaluate our hypothesis about a relative task-initial unity developing towards diversity, we first ran a series of models that constrained the within-time correlation among all the paradigm-specific latent factors to 1.00 at each timepoint and used a nested χ^2^ difference test to evaluate whether these constraints caused the model to fit significantly worse than the initial unconstrained model. Starting with the initial time point, the constrained model did not fit significantly different when compared with the baseline strong model (Δχ^2^ = 0.069, Δ*df* = 3, *p* = .995), indicating that at the first time point, the paradigm-specific latent factors were not differentiated. Keeping these constraints, we applied the unity constraints also to the second time point and found that this model fit was significantly worse than the baseline model (Δχ^2^ = 29.718, Δ*df* = 6, *p* < .001), as was the case at the third (Δχ^2^ = 51.112, Δ*df* = 8, *p* < .001) and fourth (Δχ^2^ = 54.454, Δ*df* = 10, *p* < .001) timepoints. In other words, the latent factors behaved as a single construct at the first timepoint but not after that.

Next, we examined in more detail how the latent factor variances and covariances behaved over time by constraining them one by one, that is, for each of the paradigm-specific latent factors separately and not all at the same time as was done above. We evaluated whether our paradigm-specific latent factors differentiated at any single time point by constraining their correlations to be 1.00 at that time point and conducting a nested-model χ^2^ test to see whether this constraint worsens model fit. With the same method, and building on the model where the constraints to 1.00 were made when possible, we also examined whether the intercorrelations of the paradigm-specific latent factors could be constrained to be equal (irrespective of their strength) across time points. Failure to do so would indicate that the correlations change over time. To evaluate correlations on the same scale, rescaling constructs were used to estimate standardised covariances among all latent constructs (Little et al., 2006). Rescaling constructs consist of a standardised latent construct that is modelled with a single factor loading onto the original unscaled construct, and all covariance relationships among the unscaled constructs are moved to be between the standardised scaled constructs. This allows for the variance of the unscaled constructs to be estimated on the factor loading, and all the covariance relationships among the rescaling constructs to be estimated in a correlational metric that can be compared across the different time points.

After these operations, the final constrained values for the variances and intercorrelations of our latent parameters can be found on Table 2. In this table, an asterisk (*) indicates that a parameter has been constrained to be equal to 1.00 or to another parameter (irrespective of the strength of the correlation) within a task phase, and a cross (†) indicates that a parameter has been constrained to be equal with another parameter (irrespective of the strength) over task phases. Based on our hypothesis, we expected the final model to show that at the initial task phase or phases, the paradigm-specific factors can be constrained to correlate at one and behave as a single construct, and then differentiate over time, indicated by a failure to constrain the correlations to be equal across task phases. In addition, correlations were constrained to be equal to another within a task phase (irrespective of strength) to indicate when the correlations were equidistant, and to see whether a relationship that was constrained to be equal at one task phase is no longer equal at another task phase. As noted above, this was also done to attain the most parsimonious model where the number of parameters to be estimated is minimised, thus increasing statistical power.

**Table 2.** Experiment 1: Final parameter estimates.

Correlations												
	NB T1	RM T1	SP T1	NB T2	RM T2	SP T2	NB T3	RM T3	SP T3	NB T4	RM T4	SP T4
NB T1	1.000											
RM T1	1.000*	1.000										
SP T1	1.000*	1.000*^†^	1.000									
NB T2	1.010	0.868	0.804	1.000								
RM T2	0.819	1.077	0.957	0.721*^†^	1.000							
SP T2	0.832	0.919	1.054	0.721*^†^	1.000*^†^	1.000						
NB T3	1.002	0.766	0.724	1.010	0.768	0.685	1.000					
RM T3	0.784	1.309	0.842	0.698	1.136	0.868	0.721*^†^	1.000				
SP T3	0.881	1.028	1.054	0.764	1.146	1.011	0.721*^†^	1.000*^†^	1.000			
NB T4	0.995	0.764	0.707	0.997	0.668	0.678	0.994	0.674	0.710	1.000		
RM T4	0.839	0.966	0.830	0.722	1.175	0.988	0.725	0.908	0.850	0.721*^†^	1.000	
SP T4	0.973	1.055	1.030	0.808	0.919	1.022	0.728	0.908	1.079	0.721*^†^	0.721*	1.000
*M*	1.631	2.383	0.689	2.508	2.433	0.675	2.700	2.562	0.670	2.795	2.522	0.663
*SD*	0.147	0.493^†^	0.128^†^	0.614	0.493^†^	0.128^†^	0.841	0.493^†^	0.128^†^	0.948	0.493^†^	0.128^†^

Parameters marked with an asterisk (*) are constrained to be equal to one or another parameter within time, and parameters marked with a cross (†) are constrained across time. As a rule of thumb, the absence of cross symbol indicates a significant change between the time points, that is, in those case constraining the factor correlations to be equal over time has resulted in significantly worse model fit.

NB: *n*-back; RM: running memory; SP: span; T = task phase.

These analyses showed, in line with the first analysis, that all three paradigm-specific constructs were undifferentiated at the first task phase, as all the factor correlations could be constrained to one without worsening model fit. *Running memory* and *span* continued to be undifferentiated at the second and third task phases, and *n-back* showed a strong but below 1 correlation with this combined construct at second (*r* = .721) and third task phases (*r* = .721). That is, at these task phases, *n-back* was no longer unitary with *running memory* and *span*, as constraining *n-back* to fully correlate with the two other tasks led to a significantly worse model fit. However, although all paradigm-specific constructs remained highly correlated by the fourth time point, they demonstrated differentiation by no longer having a correlation of 1.00. Instead, all of them were correlated with each other at *r* = .721. In other words, at this final task phase, constraining the correlations to 1.00 led to a significantly worse model fit, indicating that the latent factor structure could no longer be considered unitary. This change from relative unity towards paradigm-specific diversity mainly took place at the very early phases of task performance. Only the correlation between *running memory* and *span* remained static at one until the third time point, whereas all other correlations significantly decreased from one already at the second time point. After the initial drop in the latent factor intercorrelations, most remained stable at .721 at the subsequent time points. Fit indices for the model with these correlation constraints are reported in Table 1 (see the row “Lt. Correlation”).

We also evaluated construct variances over time using this same method to attain the most parsimonious model of the variances and covariances possible. We determined that the variance (i.e., standard deviation) of *running memory* and *san* did not change over time, with *running memory* having a variance of 0.493 and *span* having a variance of 0.128 at all four task phases. The *n-back* construct, however, demonstrated a pattern of increased variance over time, with variances of 0.147, 0.614, 0.841, and 0.948, respectively. This indicates increasingly divergent performances within this particular paradigm, presumably due to the fact that it is adaptive, in contrast to the other WM task paradigms employed here. The variances and latent mean scores are summarised in Table 2. Fit indices for the model with these variance restrictions in place are reported in Table 1 (see the row “Lt. Variance”).

The final model was constructed with both the variance and covariance restrictions in place and demonstrated good overall model fit, with a χ^2^ of 795.043 (*df* = 673), *p*-value of .001, CFI of 0.976, TLI of 0.973, SRMR of 0.052, and RMSEA of 0.025 with a 90% confidence interval of [0.017, 0.031] (see Table 1, row “omnibus”). A nested χ^2^ test comparing this constrained model to the unconstrained strong invariance model demonstrated that this final model did not fit significantly worse than the unconstrained strong invariant model, Δχ^2^ = 16.058, Δ*df* = 17, *p* = .520. This indicates that our final model, being constrained to reflect the relative unity-towards-diversity pattern in factor intercorrelations that our hypothesis predicted, provided an equally good fit to the data as the unconstrained model. A full table of the nested-model test results can be found in Table 1, and the final constrained values for the variance and correlation of our latent parameters can be found in Table 2. All the factor loadings over time are presented in Supplementary Appendix B1, and the unconstrained latent factor correlation matrix in Supplementary Appendix C1.

### Discussion

According to the cognitive skill learning framework (Chein & Schneider, 2012), task-general metacognitive processes such as strategy generation and executive control should play a central role at the initial phases of complex task performance, whereas at later phases, task performance becomes partially automatised through gradually emerging task-specific subroutines. In line with this account, the present results indicated a pattern of relative unity-towards-diversity: WM task performances at the initial phase could be modelled as a single general factor, but then they started to diverge into factors specific to the task paradigm. This change in the latent factor structure challenges the common assumption that complex cognitive tasks measure static constructs throughout the task performance.

## Experiment 2

In an attempt to replicate the analyses with another data set, we ran them with the pretest results from a more recent WM training study of our research group. The data and the scripts used in the analyses is available at https://osf.io/gvqhu/.

### Participants and procedure

The data of Experiment 2 stems from the pretest of a randomised controlled online trial on WM training by Ritakallio et al. (2022). The study was approved by the Institutional Review Board of the Departments of Psychology and Logopedics, Åbo Akademi University, and it was conducted in accordance with the Helsinki Declaration. All participants gave their written informed consent. As with Experiment 1, this study was preregistered (https://osf.io/c9ygt) but because the data were collected for other purposes, the present hypothesis was not included in the preregistration.

Following an extensive two-step prescreening procedure, 250 participants were invited to take part in the study through Prolific Academic (https://www.prolific.co/). Of those, 34 participants did not complete the entire pretest, and an additional 15 participants reported cheating during task performance. This resulted in a final sample size of 201 participants who met our inclusion criteria, which were similar to those in Experiment 1: English native speakers, no current psychiatric or neurological illnesses that affected the participant’s daily life, no current use of CNS medication and no current psychotropic drug use except for tobacco, alcohol, and cannabis. The participants were 18 to 50 years old with a mean age of 32.09 (*SD* = 8.27) and 56.5% (*n* = 114) of them were females. The mean length of education was 16.13 years (*SD* = 3.35).

### Materials

The WM tasks included in the pretest battery encompassed three *n*-back tasks (digits, letters, and colours), two running memory tasks (letters and colours), two simple span tasks (letters and colours), and two SU tasks (digits and colours).

#### *n*-Back paradigm

The three task variants had digits (NBD), letters (NBL), or colours (NBC) as stimuli. Stimulus presentation and task length was the same as in Experiment 1. As in Experiment 1, there were no practice rounds.

#### Running memory

The two running memory task variants had letters (RML) and colours (RMC) as stimuli. Both variants had a stimulus presentation time of 1000 ms and an interstimulus interval of 500 ms. For both RML and RMC, the sequence lengths varied between 4 and 11 items. The sequences were presented in a randomised order, with one trial for each sequence length. Each task took ca. 5 minutes. The practice rounds were analogous to the running memory tasks in Experiment 1.

#### Span

There were two forward simple span tasks—one with letters (FSL) and the other one with colours (FSC). In both variants, the participants were presented with an item sequence (exposure time 1000 ms, interstimulus interval 500 ms), and they were to recall the items in the order of presentation. The sequence lengths varied between 4 and 9 in the FSL and 4 and 10 in the FSC, with the order of the sequences being randomised. There was one trial per sequence length. Task length was about 5 min for each variant. The practice trials were analogous to the span tasks in Experiment 1.

#### Su

The SU paradigm was based on Murty et al. (2011). As its name implies, Murty and colleagues designed this task to tap the updating component. Rather than doing it in an all-or-none fashion, SU requires the overwriting of some items in a stimulus list while maintaining other ones. Two task variants were employed—one with digits 1 to 9 (SUD) and the other one with the colours blue, yellow, red, green, purple, black, pink, orange, and grey (SUC). In other respects, SUD and SUC were identical. In both variants, five items were shown on the computer screen in a row of five boxes, and the participants were instructed to memorise the sequence. After this, the initial sequence disappeared and a new row of five boxes was shown. In the new row, two of the boxes had new items, whereas the remaining three were empty. The participants were to update the memorised sequence with the two new items while maintaining the unchanged items in their WM. In both task variants, the participants completed 10 baseline trials with no updating, and 10 trials with three updating phases where old items were replaced by new ones. At the end of each trial, the participants were to report the final item sequence. The presentation order of the trials was randomised, and the participants did not know whether the next trial sequence would be a baseline trial or entail updating. The exposure time of the initial item sequence was 4000 ms for the SUD and 7000 ms for the SUC. This was followed by a 100-ms blank screen, after which the first updating phase was presented for 2000 ms (SUD) or 5000 ms (SUC). This was again followed by a 100-ms blank screen and the next update. At the end of a trial, a recall grid with horizontally aligned boxes containing the digits 1 to 9 or the nine colours was shown on-screen. The participants were to click on the numbers or colours in the order that corresponded to the final item sequence. Each task variant took ca. 8 minutes. The practice trials were analogous to those of the span tasks. There were three to seven practice trials consisting of alternately presented updating and baseline trials. The first updating sequences were easy, with the updating locations being mainly in the beginning or at the end of the stimulus sequence. With correct recall of two consecutive easy sequences, the participants completed a more difficult trial with the updating locations mainly in the middle of the item sequence. After that, the participants proceeded to the actual task, irrespective of whether they recalled the more difficult sequence correctly. For further task details, see Fellman et al. (2018) and Laine et al. (2018).

### Dependent variables

As in Experiment 1, we split the sequences into four phases, roughly matched by length. The number of blocks in the phases was as follows: 3/3/3/3 in the *n*-back tasks (NBD, NBL, NBC); 1/2/2/2 in the running memory tasks (RML, RMC), 1/1/2/2 in the span tasks (FSL, FSC), and 2/2/3/3 in the SU tasks (SUD and SUC). The dependent variable for the *n*-back tasks was the average *n*-back level achieved in the respective task phase. For the span tasks, the dependent variable was the proportion of correctly recalled items in correct serial order, that is, the number of correctly recalled items/total number of items. For the running memory tasks and the SU tasks (for the latter one only the updating trials), the dependent variable was calculated by counting the correctly recalled items in correct position in a sequence, and then averaging the scores over sequences within the respective task phases.

### Analytical approach

We employed the same analysis method and fit indices criteria as in Experiment 1.

### Results

The data were screened for univariate outliers (i.e., scores deviating >3.5 *SD*s from the sample mean on each task); task performances exceeding this threshold were treated as missing. Moreover, performances from participants who reported being colour-blind were treated as missing in the colour tasks. Missingness across all time points ranged from zero to six observations (3%) for any one variable. Descriptive statistics and pairwise correlations between tasks are summarised in the Online Supplementary Materials, Appendix D.

The first analytical aim was to establish factorial invariance. Our initial CFA configural model for Experiment 2 demonstrated good fit based on our established criteria with a χ^2^ of 531.918 (*df* = 432), *p*-value of .001, CFI of 0.975, TLI of 0.964, SRMR of 0.053, and RMSEA of 0.034 with a 90% confidence interval of [0.023, 0.043]. For this measurement model, reliability for the *n-back* over the four task phases was 0.74, 0.63, 0.79, and 0.84. The corresponding reliabilities for *span* were 0.27, 0.49, 0.46, and 0.41, for *SU* 0.56, 0.51, 0.73, 0.70, and for *running memory* 0.19, 0.34, 0.48, and 0.55. As before, despite low reliabilities, all factor loadings were significant, allowing comparisons in the disattenuated latent space (Little et al., 1999). This model was evaluated for weak invariance by constraining the item loadings to be equal across time; however, this model demonstrated too great a change in CFI (ΔCFI = 0.04). We then fit a partial weak invariant model by allowing the loadings for *n-back* to be freely estimated at the initial measurement phase but constraining all subsequent task phase loadings to be equivalent. This partially invariant model passed our criteria with a change in CFI of −0.001. We then evaluated strong invariance by constraining the item intercepts to be equivalent over time; however, this model also demonstrated too great a change in CFI compared with the partial weak model (ΔCFI = 0.03). After evaluating our item intercepts, we allowed the intercepts for *n-back* at the initial phase to be freely estimated while continuing to constrain all subsequent intercepts to be equivalent over time. The resulting strong invariance model demonstrated good model fit with a χ^2^ of 547.879 (*df* = 446), *p*-value of .001, CFI of 0.975, TLI of 0.964, SRMR of 0.049, and RMSEA of 0.034 with a 90% confidence interval of [0.023, 0.043]. Fit indices for all levels of invariance can be found in Table 3.

**Table 3.** Experiment 2: Measurement invariance and nested-model comparisons.

	χ^2^	*df*	*p*	CFI	TLI	RMSEA	SRMR	ΔCFI	Δχ^2^	Δ*df*	*p*	Pass?
Configural	531.918	432	.001	0.975	0.964	0.034	0.053	–	–	–	–	Yes
Weak	709.532	435	.000	0.932	0.901	0.056	0.113	0.043	177.615	3	.000	No
Weak Partial	528.366	433	.001	0.976	0.966	0.033	0.051	0.001	−3.551	1	1.000	Yes
Strong	658.934	448	.000	0.948	0.926	0.048	0.083	0.029	130.567	15	.000	No
Strong Partial	547.879	446	.001	0.975	0.964	0.034	0.049	0.002	19.513	13	.108	Yes
Lt. Correlation	555.283	462	.002	0.977	0.968	0.032	0.052	–	7.404	16	.965	Yes
Lt. Variance	552.829	453	.001	0.975	0.966	0.033	0.051	–	4.950	7	.666	Yes
Omnibus	563.621	470	.002	0.977	0.969	0.031	0.056	–	15.741	24	.897	Yes

Measurement invariance was evaluated using the ΔCFI with .01 as the threshold for passing. Latent parameters were tested using nested model χ^2^ difference tests with an alpha of .05. “Configural,” “Weak,” “Weak Partial,” “Strong,” and “Strong Partial” refer to the models testing the respective types of invariances; “Lt. Correlation” refers to the model where the factor correlations have been constrained to 1.00 or equality, when that was possible without reducing model fit; “Lt. Variance” refers to the model where the latent score variances were constrained to equality, when that was possible without reducing model fit; and “Omnibus” refers to the model where both the correlations and variances have been constrained.

In line with Experiment 1, we first constrained all the latent factor correlations to 1.00 at each task phase and examined whether this worsens model fit. The constrained model showed worse model fit compared with the baseline model already at the first task phase (Δχ^2^ = 77, Δ*df* = 8, *p* < .001). The constrained model failed to converge at the second task phase and showed worse fit at third (Δ*χ*^2^ = 131.84, Δ*df* = 15, *p* < .001) and fourth phases (Δχ^2^ = 143.78, Δ*df* = 17, *p* < .001). This indicates that the latent factors behaved as separate constructs at all timepoints. Thus, the present data failed to replicate the initial unity of the latent paradigm-specific factors that we observed in Experiment 1.

Next, we examined in more detail how the variances and covariances of our paradigm-specific latent factors behaved over time by constraining the factor correlations one by one and not all at the same time as was done above. Thus, in line with Experiment 1, we evaluated whether the paradigm-specific factors were undifferentiated at any single time point by constraining their correlations one by one to be 1.00 and conducting a nested-model χ^2^ test to see whether the constraint worsens model fit. Next, we examined whether it is possible to constrain the intercorrelations of the factors to be equal across time to see whether there are indications of a change in the latent factor structure from unity to diversity. Finally, as in Experiment 1, factor intercorrelations within a task phase were constrained to be equal when possible to ease interpretation and to make the model as parsimonious as possible. To evaluate correlations on the same scale, rescaling constructs were once again used to estimate standardised covariances among all latent constructs (Little, 2013).

These stepwise analyses showed that at the initial task phase, the paradigm-specific factors *running memory, span*, and *SU* were behaving as a singular construct with correlations that could be constrained to one, but with *n-back* weakly correlated with *running memory* (*r* = .198) and moderately correlated with *span* and *SU* (*r* = .456). Thus, at the first task phase, all the latent factors except for *n-back* could be constrained to correlate at 1.00 without worsening model fit. At the second task phase, *running memory* and *SU* were still undifferentiated with a correlation that could be constrained to one, whereas the correlations had become moderate among *span* and *running memory* (.544) and among *span* and *SU* (.457). In other words, at the second task phase, no other paradigm-specific factors besides *running memory* and *SU* could be constrained to correlate at 1.00 without worsening model fit. By the third task phase, the constructs had completely differentiated, with the correlations between *running memory* and *span*, and *running memory* and *SU* being .781, the correlation between *SU* and *span* being .610, and all correlations between *n-back* and the other constructs being estimated at .457. At the final task phase, the constructs were still differentiated with the correlations among *SU*, *span*, and *running memory* being .781, and the correlation with all three of these constructs with *n-back* being .457. Thus, both at the third and the fourth task phases, no factors could be constrained to correlate at 1.00 without worsening model fit, suggesting a change from (partial) unity towards diversity. The exception was the *n-back* factor that exhibited no clear pattern in the evolvement of correlations with the other paradigm-specific factors. As for the other factors, after the initial drop in the factor intercorrelations from the first to the second task phase, the correlations mainly did not change between the third and fourth task phases. This indicates that the latent factor structure became more stable after the initial drop in the intercorrelations, as shown in Table 4. Fit indices for the model with all the correlation constraints are reported in Table 3 (row “Lt. Correlation”).

**Table 4.** Experiment 2: Final parameter estimates.

Correlations																
	NB T1	RM T1	SP T1	SU T1	NB T2	RM T2	SP T2	SU T2	NB T3	RM T3	SP T3	SU T3	NB T4	RM T4	SP T4	SU T4
NB T1	1.000															
RM T1	0.198	1.000														
SP T1	0.456*	1.000*	1.000													
SU T1	0.456*	1.000*^†^	1.000*	1.000												
NB T2	1.087	0.186	0.451	0.592	1.000											
RM T2	0.295	1.328	0.881	0.954	0.317*	1.000										
SP T2	0.305	0.704	0.912	0.578	0.317*	0.544*	1.000									
SU T2	0.442	0.962	0.969	1.095	0.544*	1.000*^†^	0.457*	1.000								
NB T3	1.128	0.196	0.351	0.456	0.989	0.272	0.293	0.462	1.000							
RM T3	0.472	1.021	0.893	0.868	0.518	1.184	0.600	0.868	0.457*^†^	1.000						
SP T3	0.448	0.965	0.895	0.791	0.532	0.753	0.606	0.629	0.457*^†^	0.781*^†^	1.000					
SU T3	0.447	0.726	0.862	1.071	0.510	0.807	0.434	1.147	0.457*^†^	0.781*^†^	0.610	1.000				
NB T4	1.162	0.177	0.420	0.441	0.984	0.265	0.313	0.474	1.006	0.463	0.433	0.454	1.000			
RM T4	0.450	1.063	0.637	0.962	0.455	1.029	0.500	0.861	0.474	0.826	0.472	0.789	0.457* ^†^	1.000		
SP T4	0.465	1.116	0.966	1.075	0.550	1.099	0.999	1.006	0.452	0.937	1.078	0.853	0.457* ^†^	0.781* ^†^	1.000	
SU T4	0.443	0.769	0.735	1.070	0.478	0.918	0.405	1.076	0.458	0.825	0.628	1.032	0.457* ^†^	0.781* ^†^	0.781*	1.000
*M*	1.836	2.670	0.644	3.025	2.494	2.790	0.635	3.040	2.860	2.776	0.633	3.056	2.990	2.760	0.636	3.087
*SD*	0.324	0.581^†^	0.138^†^	0.716^†^	0.493	0.581^†^	0.173	0.716^†^	0.875	0.581^†^	0.138^†^	0.876^†^	1.060	0.581^†^	0.138^†^	0.876^†^

Parameters marked with an asterisk (*) are constrained to be equal to one or another parameter within time, and parameters marked with a cross (†) are constrained across time. As a rule of thumb, the absence of cross symbol indicates a significant change between the time points, that is, in those case constraining the factor correlations to be equal over time has resulted in significantly worse model fit.

NB: *n*-back, RM: running memory; SP: span, SU: selective updating, T: task phase.

To attain the most parsimonious final model, we also evaluated construct variance over time using this same method and determined that the variance (i.e., standard deviation) of *running memory* was 0.581 over all task phases. The variance of *span* was 0.138 at the first, third, and fourth task phase and 0.173 on the second. The variance of *SU* was 0.716 for the first two task phases and 0.876 on the last two phases. The variance of *n-back* increased at every task phase, with the first one being 0.324, followed by 0.493, 0.875, and finally 1.060. The latent construct means are reported in Table 4. Again, it was only the adaptive task paradigm (*n*-back) that showed increasingly diverging performances over the test session. Fit indices for the model with these variance restrictions are shown in Table 3 (“Lt. Variance”).

The final model was constructed with the variance and covariance restrictions in place, and it demonstrated good overall model fit, with a *χ*^2^ of 563.621 (*df* = 470), *p*-value of .002, CFI of 0.977, TLI of 0.969, SRMR of 0.056, and RMSEA of 0.031 with a 90% confidence interval of [0.020, 0.041]. A nested χ^2^ test comparing this model to the strong invariance model demonstrated that this final model did not fit significantly worse than the unconstrained strong invariant model, Δχ^2^ = 15.741, Δ*df* = 24, *p* = .897. This indicates that constraining the correlations to manifest the unity-towards-diversity pattern in the way described above fits the data equally well as the unconstrained model. A full table of nested-model test results can be found in Table 3, and the final constrained values for the variance and correlation of our latent parameters can be found in Table 4. All the factor loadings over time are presented in Supplementary Appendix B2, and the unconstrained correlation matrix can be found in Supplementary Appendix C2.

### Discussion

Experiment 2 partly replicated the results of Experiment 1 with new participants and with a slightly different set of WM tasks. The most clearcut difference was that the latent factors could not be fitted with a model exhibiting unity at the first timepoint. The detailed analysis revealed that this was due to the *n*-back task being separated from the other WM task paradigm factors, whereas those other factors indicated relative task-initial unity. In other respects, the results were in line with the relative unity-towards-diversity hypothesis, with diversity of the latent paradigm-specific factors increasing over time.

One potential reason for this difference in the outcomes of Experiments 1 and 2 may lie in the tasks. In Experiment 1, each task type included spatial stimuli, but in Experiment 2, no spatial stimuli were included. There is evidence that spatial tasks rely more on executive processes than do verbal tasks, which could be reflected as higher task-initial latent factor intercorrelations in Experiment 1 (Miyake et al., 2001). However, this does not imply that the general factor would be solely driven by the spatial tasks in Experiment 1, because that would have led to a failure to find any unity in Experiment 2 which did not include spatial tasks. Instead, inclusion of the spatial tasks could have enhanced initial task loadings on general executive processes in Experiment 1.

## Post hoc analyses

### Analyses without the *n*-back task

A general issue with both experiments is that, unlike the other WM tasks, *n*-back was adaptive, with the difficulty level being adjusted depending on the performance level of the participant. It is unclear how this might have affected the results. Thus, we re-ran the analyses by constraining the latent factor correlations one-by-one as reported above, but without the *n*-back task.

In the case of Experiment 1, the original results were not fully replicated, and the remaining tasks running memory and span showed a pattern of correlation from .898 through 1.0 in the second and third task phases to .806 in the last task phase. Thus, although the correlations were strong at the initial task phases and weakest (but still strong) at the last task phase, in line with their expected attenuation over time, they could not be constrained to perfect unity at the initial phase. However, in Experiment 2, where the original analyses including the *n*-back factor failed to show a fully consistent relative unity-to-diversity pattern, task-initial unity was observed when *n*-back was left out of the analysis. All the remaining tasks *running memory*, *span* and *SU* could be constrained to correlate at one at the initial task phase, but at the second phase, the correlation between *running memory* and *span* dropped to .417 and between *span* and *SU* to .449. At the third and fourth task phases, all constructs had become differentiated with moderate-to-strong correlations. Full tables of nested-model test results as well as the final constrained variance and correlation matrices of the latent parameters for both experiments without *n*-back can be found in Supplementary Appendix E.

### Testing alternative models

A potential issue with the present analytical approach is that there can be several alternative models that fit the data equally well. For example, our approach does not consider a model where the latent factor correlations are equal between the time points, whatever their estimated value may be. Note that such a model would not address our relative unity-towards-diversity hypothesis, but it could be considered as one possible competing model that reflects a static view of the mental architecture underlying task performance. Thus, we analysed whether it is possible to constrain all the covariances to equality between all the task phases (equality model), whatever the strength of the correlations would be. This model did not produce a significant misfit as compared with the strong invariant model in Experiment 1 (Δχ^2^ = 10.149, *p* = .339, CFI = .976, TLI = .972, RMSEA = .025, SRMR = .051) or in Experiment 2 (Δχ^2^ = 23.712, *p* = .165, CFI = .973, TLI = .964, RMSEA = .034, SRMR = .052). In Experiment 1, the latent factor intercorrelations estimated in this way were between .693 and .934. In Experiment 2, they were between .603 and .741 for all the other tasks except for *n*-back, where they were between .421 and .496.

Given the adequate fit for the Equality model, we also tested a further model that constrained covariances to unity at the first time point and to equality at the following time points. This Unity to Equality model can be considered as a variant of our relative unity-towards-diversity hypothesis. For the first experiment, the Unity to Equality model did not fit significantly worse than the unconstrained model (Δχ^2^ = 3.262, *p* = .953, CFI = .977, TLI = .973, RMSEA = .024, SRMR = .050), unlike in the second experiment where misfit was observed (Δχ^2^ = 88.204, *p* = .000, CFI = .957, TLI = .942, RMSEA = .043, SRMR = .070).

The two post hoc models cannot be directly contrasted as they are not nested, but we may compare their fit indices. In the first experiment, both the Equality model and the Unity to Equality model showed adequate fit, but the misfit was less for the Unity to Equality than for the Equality model, as indicated by better fit indices. The Unity to Equality model had better (lower) Akaike information criterion (AIC) and Bayesian information criterion (BIC) values (AIC = 15396.488, BIC = 16116.108) than the Equality model (AIC = 15403.376, BIC = 16122.996). This is in line with our hypothesis. In contrast, in the second experiment, the Unity to Equality model showed misfit unlike the Equality model, which could be taken to reflect the fact that for this experiment, task-initial unity was only partial, as described in 3.5 above.

## General discussion

This study addressed a fundamental yet mostly implicit assumption in cognitive testing, namely that the cognitive processes underlying complex task performance remain static throughout the whole task. In contrast, the cognitive skill learning view implies that performing a task is an adaptive learning process that proceeds from a heavy engagement of task-initial general metacognitive and executive control processes towards more automatic and task-specific ones (Ackerman, 1988; Chein & Schneider, 2012; Schneider & Chein, 2003; Schneider & Shiffrin, 1977; Shiffrin & Schneider, 1977). Here, we applied this approach for the first time to widely used WM tasks by examining possible latent structure changes during task performances that take only a few minutes. The results of the main analyses from the two independent data sets in Experiments 1 and 2 were for the most part consistent with our relative unity-towards diversity hypothesis. The only exception to this pattern was *n*-back in Experiment 2 that differentiated from the other factors already at the initial task phase.

Thus, in line with the cognitive skill learning framework, the findings of our main analyses suggest a change from a high task-initial engagement of general metacognitive and executive control processes towards more task-specific ones later on. It appears that in the present WM tasks, the initial high engagement of general resources is quite short-lived, as the paradigm-specific factors could be constrained to unity only at the initial task phase, showing diversification thereon. This rather fast change is supported by independent evidence from previous microgenetic studies that focused on spontaneous strategy use in different memory tasks (Laine, Fellman, et al., 2024; Laine, Jylkkä, et al., 2024; Waris, Fellman et al., 2021; Waris, Jylkkä et al., 2021). These studies show that many participants generate and use strategies right from the first task block and the rates of changes in strategies are highest between the first two to three blocks of a task, suggesting intensive engagement of metacognitive and executive control at the task-initial phases. Thereafter, use of memory strategies becomes more stable while performance is steadily enhanced. This could reflect increased routine in the employment of the strategies in a specific memory task.

The results of our main analyses can be compared with earlier studies that have examined WM latent factor intercorrelations in the context of WM training (De Simoni & von Bastian, 2018; Meiran et al., 2019). De Simoni and von Bastian observed a pre-post decrease in the correlation between their WM-related latent factors binding and updating (from .91 to .75; see Figure 3 in their paper). Meiran and colleagues analysed training session data from the experiment of De Simoni and von Bastian by looking into the change of shared variance across the training sessions within the groups that trained binding and updating tasks, respectively. More specifically, they analysed the similarity of the rank-ordering of the individual differences on their four binding or updating training tasks in each session. Their principal components analysis showed a gradual increase of the eigenvalue of the first component, indicating increased similarity of performance as the training progressed (binding tasks for one training group, updating tasks for another). These results would thus suggest that repeated practice leads to a more similar processing within a WM task paradigm, while processing between task paradigms diverges over time. However, it must be emphasised that these studies are not directly comparable to ours, as their time scale was very different. It spanned several weeks, whereas our time scale was at the level of minutes.

### Findings of the post hoc analyses

In our analyses, the adaptive nature of the *n*-back task could have resulted in it behaving differently from the other tasks. Thus, a post hoc analysis was conducted without the *n*-back task. In Experiment 1, this resulted in failure to replicate the relative unity-towards-diversity pattern, although the remaining task paradigm factors were still highly correlated and the correlation was lowest at the last task phase. In contrast, in Experiment 2 the exclusion of the *n*-back task resulted in the relative unity-towards-diversity pattern. This suggests that the present kind of analysis is sensitive to the tasks included and calls for replications with other sets of tasks.

We also examined two alternative models not specified in our initial hypothesis, namely a model where the latent factor intercorrelations were constrained to equality (whatever their strength) between all the task phases (the Equality model) and a model where the intercorrelations were constrained to unity at the first stage and equality at subsequent phases (the Unity to Equality model). Although the Equality model does not address our hypothesis, it could be taken to reflect the theoretical view where the cognitive processes involved in task performance are static over time. The Unity to Equality model, in turn, can be considered as a variant of our unity to diversity hypothesis. The Equality model did not show a significantly worse fit in either of our experiments. For Experiment 1, it did fare worse than the Unity to Equality model, but the opposite was true for Experiment 2 where only the Equality model showed adequate fit. The latter fact could be taken to reflect the finding of the main analysis that the task-initial unity in Experiment 2 was only partial, with *n*-back behaving differently than the other tasks. All in all, as both unity-towards-diversity (with full or partial unity) and equality-across-time models fit with the present data sets, one can conclude that while attenuation of the latent factor intercorrelations across the time does take place as our hypothesis states, this attenuation is limited. The overall high latent factor intercorrelations also reflect the fact that all our tasks represented a single cognitive domain, WM. The relative unity-towards-diversity hypothesis is not tied into WM domain only, and it is possible that this pattern, if replicated, would be more evident with a set of executive tasks that represent multiple executive task domains.

### Limitations

An important limitation is that our analytical method (LSEM) can only yield indirect evidence of the processes underlying task performance. Based on the cognitive skill learning framework, we assume that a relative task-initial unity in the latent factors reflects a strong engagement of metacognitive and executive control processes, whereas a gradually emerging diversity reflects a shift towards more automatic, task-specific lower-level processes (Chein & Schneider, 2012; Taatgen, 2013). Confirming this presumption would benefit from more direct measures of the underlying processes. As to the hypothesised buildup of partial automaticity, one possibility could be to follow-up the speed of the basic stimulus-response mapping required to perform the task. To take a concrete example, in an experiment consisting of adaptive *n*-back tasks, one could employ intermittent 1-back blocks that have a minimal WM load and track whether the reaction times are speeded up over the course of the experiment. This could tap on the emergence of task-specific automatic production rules at the sensory-motor level (Taatgen, 2013). Another possible option would be to use a simple intermittent secondary task. Due to the hypothesised initial high executive load on the complex and unfamiliar primary task, the secondary task should create clear interference early on. However, this interference effect should be attenuated over time, reflecting increase in executive resources that are freed for the secondary task.

Another potential limitation relates to probable intertask and interindividual differences in when the peak executive load occurs. To take an example of the former, in simple span tasks, executive load can be expected to be weaker than in more complex WM tasks and arguably peaks at an early stage, whereas more complex WM tasks assumedly load on the executive system for a longer period. The length and intensity of executively taxing task-initial periods may also vary considerably between participants. The current analytical method with rough splits of the task periods at a group level does not fully take these factors into account. This problem is augmented by the existence of customary practice trials (the number of which depends on individual performance) for the nonadaptive WM tasks. Hypothetically, the existence of practice trials should lead to a buildup of task-specific routines earlier on and should work against our hypothesis, leading to an underestimation of the relative task-initial unity. Multilevel methods where individual slopes for tasks and participants can be specified could aid in detecting such effects, but to our knowledge, this is not possible in latent factor analyses. A related limitation is that the time resolution in our analysis is somewhat coarse. This limitation is due to the use of SEM, which is based on individual differences and requires a relatively large number of observations per time point.

A technical limitation of this study pertains to reliability which appeared to be poor for several of the constructs at various time points across both experimental conditions, as assessed using McDonald’s omega coefficient. However, while reliability can be informative of the performance of the indicators of a construct, the reliability of the indicator does not dictate its viability for measuring an underlying construct in an SEM framework (Little et al., 1999). Simulation studies have demonstrated that items with poor psychometric properties such as low reliability can produce accurate estimates of the underlying variance and covariance structure of the latent constructs as long as they sufficiently cover the underlying construct space, yield sufficient variability on the construct as demonstrated by the significance of their loadings, and are analysed in the context of SEM to accurately model and disassociate the reliable variance from the unreliable variance (Little et al., 1999). There was indeed variability, and the loadings were significant, but it could still be argued that the small number of observations per time point in this study poses the risk of not sufficiently covering the construct space.

The tasks of this study stem from previous cognitive training studies designed for other purposes, and at least, some of the tasks may not be ideal for the present type of analysis. For example, the present *n*-back tasks were adaptive which lessens the degree to which they can be automatised, there were practice rounds that decreased task novelty prior to the initial measurement phase, and span length variation between trials in the span tasks added variability to the results. Moreover, the length of the tasks varied substantially, with the *n*-back task being the longest one due to its adaptivity. These factors should be considered in future studies. The fact that the main results were nevertheless largely replicated in two independent data sets with partly different sets of tasks is nevertheless positive. Moreover, omitting *n*-back from the analyses did not systematically change the overall pattern, which became less consistent with the hypothesis in Experiment 1, but more consistent in Experiment 2.

A more specific interpretational issue is what the relative unity at the initial task phases reflects. In line with the cognitive skill learning view, we have hypothesised that it reflects heavy engagement of the metacognitive system (e.g., strategy generation, task initiation, and monitoring) and executive control (e.g., attention allocation and process sequencing for the adopted performance strategies, performance monitoring, and adjustment). It is worth noting that the unity cannot indicate fully shared cognitive processes between the tasks (hence, the use of the term “relative unity” in this article), given their structural differences and different stimulus materials, which necessitate the engagement of task-specific sensory-motor systems right from the start. Thus, a full unity at the level of cognitive processes is not what the present analyses suggest, but instead that the different tasks can be considered to form a psychometrically unitary construct. This is a general feature of the SEM method and not specific to this study. For example, verbal and spatial intelligence tasks often form a single construct (Meiran et al., 2019), but this obviously does not imply that the underlying cognitive processes would be identical.

A related question is what the paradigm-specific factors, differentiating in the subsequent task phases, are reflecting. This cannot be answered based on the present results alone, but it can be hypothesised that they implicate an increasing role of cognitive processes that are specific to the task structure. This could be explicated in terms of Taatgen’s (2013) primitive information processing elements (PRIM) model. Any task typically requires several such elements, and their consecutive employment requires executive control when a task is novel. However, through learning the elements can be clustered into “production rules” that enable more effortless and automatic performance of several processing elements in a row (see Figure 3 in Taatgen, 2013). The diversification of the latent constructs into paradigm-specific factors in this study could reflect such a process. However, the formation of the paradigm-specific factors does not imply that the processes would be identical between the task variants that form the factor or that the process would be completely automatic. The differences are gradual, not absolute, and it seems evident that a complex WM task can never become fully automatic.

Assuming that the progress from more task-general towards more task-specific processes reflects acquisition of new cognitive skills geared to the tasks, one could ask why this is not seen in increased mean performance over the four time points. A possible reason for this is a phenomenon coined as “utilisation deficiency” where participants employing a memory strategy benefit from it in their recall performance not until later. Although utilisation deficiency has been examined mostly in children, it has also been documented in adults facing a novel and demanding memory task (Gaultney et al., 2005). Implementation of newly developed strategies can initially be too effortful to facilitate performance but over time, repeated practice leads to improved memory performance, as is also evidenced by participants’ subsequent progress on the trained WM tasks in the intervention studies wherefrom our data was taken (Fellman et al., 2020; Ritakallio et al., 2022).

### Study implications

The present results, while preliminary and in need of replication, imply that the skill learning approach could be relevant also to other complex cognitive tasks than WM measures. In their highly influential factor analysis of a battery of executive function tasks, Miyake and colleagues (2000) divided executive functions into three latent factors, namely inhibition, shifting, and WM updating/monitoring. Future research should run the present type of analyses with the kinds of tasks that Miyake and colleagues used. Whereas they employed summative scores and found that the executive functions system shows both unity and diversity (in that there are separable but correlated factors), we would expect that the inclusion of the temporal dimension would reveal a shift from task-initial relative unity towards diversity as the tasks progress. If this holds, the practical implication would be that initial performances on complex tasks are better indicators of executive functioning than summative scores (for a recent attempt in this direction, see Nordenswan et al., 2020). All in all, we agree with Gonthier and Roulin (2020) that time-structured intraindividual performance variability should be taken better into account during cognitive testing. Performing a cognitive task partly creates what is being measured, as the cognitive system adapts to the test, in line with theories of situated or embodied cognition (Anderson, 2014).

## Supplemental Material

sj-docx-1-qjp-10.1177_17470218241278272 – Supplemental material for From task-general towards task-specific cognitive operations in a few minutes? Working memory performance as an adaptive process Supplemental material, sj-docx-1-qjp-10.1177_17470218241278272 for From task-general towards task-specific cognitive operations in a few minutes? Working memory performance as an adaptive process by Jussi Jylkkä, Zachary Stickley, Daniel Fellman, Otto Waris, Liisa Ritakallio, Todd D Little, Juha Salmi and Matti Laine in Quarterly Journal of Experimental Psychology

sj-docx-2-qjp-10.1177_17470218241278272 – Supplemental material for From task-general towards task-specific cognitive operations in a few minutes? Working memory performance as an adaptive process Supplemental material, sj-docx-2-qjp-10.1177_17470218241278272 for From task-general towards task-specific cognitive operations in a few minutes? Working memory performance as an adaptive process by Jussi Jylkkä, Zachary Stickley, Daniel Fellman, Otto Waris, Liisa Ritakallio, Todd D Little, Juha Salmi and Matti Laine in Quarterly Journal of Experimental Psychology

sj-docx-3-qjp-10.1177_17470218241278272 – Supplemental material for From task-general towards task-specific cognitive operations in a few minutes? Working memory performance as an adaptive process Supplemental material, sj-docx-3-qjp-10.1177_17470218241278272 for From task-general towards task-specific cognitive operations in a few minutes? Working memory performance as an adaptive process by Jussi Jylkkä, Zachary Stickley, Daniel Fellman, Otto Waris, Liisa Ritakallio, Todd D Little, Juha Salmi and Matti Laine in Quarterly Journal of Experimental Psychology

sj-docx-4-qjp-10.1177_17470218241278272 – Supplemental material for From task-general towards task-specific cognitive operations in a few minutes? Working memory performance as an adaptive process Supplemental material, sj-docx-4-qjp-10.1177_17470218241278272 for From task-general towards task-specific cognitive operations in a few minutes? Working memory performance as an adaptive process by Jussi Jylkkä, Zachary Stickley, Daniel Fellman, Otto Waris, Liisa Ritakallio, Todd D Little, Juha Salmi and Matti Laine in Quarterly Journal of Experimental Psychology

sj-docx-5-qjp-10.1177_17470218241278272 – Supplemental material for From task-general towards task-specific cognitive operations in a few minutes? Working memory performance as an adaptive process Supplemental material, sj-docx-5-qjp-10.1177_17470218241278272 for From task-general towards task-specific cognitive operations in a few minutes? Working memory performance as an adaptive process by Jussi Jylkkä, Zachary Stickley, Daniel Fellman, Otto Waris, Liisa Ritakallio, Todd D Little, Juha Salmi and Matti Laine in Quarterly Journal of Experimental Psychology

## References

[bibr1-17470218241278272] Ackerman P. L. (1988). Determinants of individual differences during skill acquisition: Cognitive abilities and information processing. Journal of Experimental Psychology: General1, 117(3), 288–318.

[bibr2-17470218241278272] Anderson M. L. (2014). After phrenology. Neural reuse and the interactive brain. MIT Press. 10.1017/S0140525X15000631 26077688

[bibr3-17470218241278272] Au J. Sheehan E. Tsai N. Duncan G. J. Buschkuehl M. Jaeggi S. M. (2015). Improving fluid intelligence with training on working memory: A meta-analysis. Psychonomic Bulletin and Review, 22(2), 366–377. 10.3758/s13423-014-0699-x 25102926

[bibr4-17470218241278272] Badre D. Kayser A. S. D’Esposito M. (2010). Frontal cortex and the discovery of abstract action rules. Neuron, 66(2), 315–326. 10.1016/j.neuron.2010.03.025 20435006 PMC2990347

[bibr5-17470218241278272] Chein J. M. Schneider W. (2012). The brain’s learning and control architecture. Current Directions in Psychological Science, 21(2), 78–84. 10.1177/0963721411434977

[bibr6-17470218241278272] Cheung G. W. Rensvold R. B. (2002). Evaluating goodness-of-fit indexes for testing measurement invariance. Structural Equation Modeling, 9(2), 233–255. 10.1207/S15328007SEM0902_5

[bibr7-17470218241278272] De Simoni C. von Bastian C. C . (2018). Working memory updating and binding training: Bayesian evidence supporting the absence of transfer. Journal of Experimental Psychology: General, 147(6), 829–858. 10.1037/xge0000453 29888939

[bibr8-17470218241278272] Fellman D. Jylkkä J. Waris O. Soveri A. Ritakallio L. Haga S. . . .Laine M. (2020). The role of strategy use in working memory training outcomes. Journal of Memory and Language, 110, 104064. 10.1016/j.jml.2019.104064

[bibr9-17470218241278272] Fellman D. Soveri A. Viktorsson C. Haga S. Nylund J. Johansson S. . . .Laine M. (2018). Selective updating of sentences: Introducing a new measure of verbal working memory. Applied Psycholinguistics, 39(2), 275–301. 10.1017/S0142716417000182

[bibr10-17470218241278272] Forsberg A. Fellman D. Laine M. Johnson W. Logie R. H. (2020). Strategy mediation in working memory training in younger and older adults. Quarterly Journal of Experimental Psychology, 73(8), 1206–1226. 10.1177/1747021820915107 PMC7575302 32160812

[bibr11-17470218241278272] Gathercole S. E. Dunning D. L. Holmes J. Norris D. (2019). Working memory training involves learning new skills. Journal of Memory and Language, 105, 19–42. 10.1016/J.JML.2018.10.003 PMC6591133 31235992

[bibr12-17470218241278272] Gaultney J. F. Kipp K. Kirk G. (2005). Utilization deficiency and working memory capacity in adult memory performance: Not just for children anymore. Cognitive Development, 20(2), 205–213. 10.1016/J.COGDEV.2005.02.001

[bibr13-17470218241278272] Gonthier C. Roulin J. L. (2020). Intraindividual strategy shifts in Raven’s Matrices, and their dependence on working memory capacity and need for cognition. Journal of Experimental Psychology: General, 149(3), 564–579. 10.1037/xge0000660 31318256

[bibr14-17470218241278272] Karbach J. Verhaeghen P. (2014). Making working memory work: A meta-analysis of executive-control and working memory training in older adults. Psychological Science, 25(11), 2027–2037. 10.1177/0956797614548725 25298292 PMC4381540

[bibr15-17470218241278272] Kassai R. Futo J. Demetrovics Z. Takacs Z. K. (2019). A meta-analysis of the experimental evidence on the near- and far-transfer effects among children’s executive function skills. Psychological Bulletin, 145(2), 165–188. 10.1037/bul0000180 30652908

[bibr16-17470218241278272] Kirchner W. K. (1958). Age differences in short-term retention of rapidly changing information. Journal of Experimental Psychology, 55(4), 352–358. 13539317 10.1037/h0043688

[bibr17-17470218241278272] Laine M. Fellman D. Eräste T. Ritakallio L. Salmi J. (2024). Strategy use and its evolvement in word list learning: A replication study. Royal Society Open Science, 11, 230651. 10.1098/rsos.230651 38356871 PMC10864779

[bibr18-17470218241278272] Laine M. Fellman D. Waris O. Nyman T. J. (2018). The early effects of external and internal strategies on working memory updating training. Scientific Reports, 8(1), 1–12. 10.1038/s41598-018-22396-5 29511316 PMC5840432

[bibr19-17470218241278272] Laine M. Jylkkä J. Ritakallio L. Eräste T. Kangas S. Hering A. . . .Salmi J. (2024). Spontaneous memory strategies in a videogame simulating everyday memory tasks. Quarterly Journal of Experimental Psychology, 77(3), 611–625. 10.1177/17470218231183958 PMC10958750 37309805

[bibr20-17470218241278272] Little T. D. (2013). Longitudinal structural equation modeling. Guilford Press.

[bibr21-17470218241278272] Little T. D. Bontempo D. Rioux C. Tracy A. (2022). On the merits of longitudinal multiple group modelling: An alternative to multilevel modelling for intervention evaluations. International Journal of Research and Method in Education, 45(5), 437–449. 10.1080/1743727X.2021.1973992

[bibr22-17470218241278272] Little T. D. Lindenberger U. Nesselroade J. R. (1999). On selecting indicators for multivariate measurement and modeling with latent variables: When “good” indicators are bad and “bad” indicators are good. Psychological Methods, 4(2), 192.

[bibr23-17470218241278272] Little T. D. Slegers D. W. Card N. A. (2006). A non-arbitrary method of identifying and scaling latent variables in SEM and MACS models. Structural Equation Modeling: A Multidisciplinary Journal, 13(1), 59–72.

[bibr24-17470218241278272] McDonald R. P. (1999). Test theory: A unified treatment. Erlbaum.

[bibr25-17470218241278272] Meiran N. Dreisbach G. von Bastian C. C. (2019). Mechanisms of working memory training: Insights from individual differences. Intelligence, 73, 78–87.

[bibr26-17470218241278272] Melby-Lervåg M. Redick T. S. Hulme C. (2016). Working memory training does not improve performance on measures of intelligence or other measures of “far transfer”: Evidence from a meta-analytic review. Perspectives on Psychological Science, 11(4), 512–534. 10.1177/1745691616635612 27474138 PMC4968033

[bibr27-17470218241278272] Melby-Lervåg M. Hulme C. (2013). Is working memory training effective? A meta-analytic review. Developmental Psychology, 49(2), 270–291. 10.1037/a0028228 22612437

[bibr28-17470218241278272] Miller E. K. Cohen J. D. (2001). An integrative theory of prefrontal cortex function. Annual Review of Neuroscience, 24(1), 167–202. 10.1146/annurev.neuro.24.1.167 11283309

[bibr29-17470218241278272] Miyake A. Friedman N. P. Emerson M. J. Witzki A. H. Howerter A. Wager T. D. (2000). The unity and diversity of executive functions and their contributions to complex “frontal lobe” tasks: A latent variable analysis. Cognitive Psychology, 41(1), 49–100. 10.1006/cogp.1999.0734 10945922

[bibr30-17470218241278272] Miyake A. Friedman N. P. Rettinger D. A. Shah P. Hegarty M. (2001). How are visuospatial working memory, executive functioning, and spatial abilities related? A latent-variable analysis. Journal of Experimental Psychology: General, 130(4), 621–640. 10.1037/0096-3445.130.4.621 11757872

[bibr31-17470218241278272] Murty V. P. Sambataro F. Radulescu E. Altamura M. Iudicello J. Zoltick B. . . .Mattay V. S. (2011). Selective updating of working memory content modulates meso-cortico-striatal activity. NeuroImage, 57(3), 1264–1272. 10.1016/j.neuroimage.2011.05.006 21596142 PMC3908780

[bibr32-17470218241278272] Nordenswan E. Kataja E. L. Deater-Deckard K. Korja R. Karrasch M. Laine M. . . .Karlsson H. (2020). Latent structure of executive functioning/learning tasks in the CogState computerized battery. Sage Open, 10(3), 1–10. 10.1177/2158244020948846

[bibr33-17470218241278272] Norman D. A. Shallice T. (1986). Attention to action: Willed and automatic control of behavior. In Davidson R. J. Schwartz G. E. Shapiro D. (Eds.), Consciousness and self-regulation (pp. 1–14). Springer.

[bibr34-17470218241278272] R Core Team. (2021). R: A language and environment for statistical computing. R Foundation for Statistical Computing. https://www.r-project.org/

[bibr35-17470218241278272] Ritakallio L. Fellman D. Jylkkä J. Waris O. Lönnroth N. Nervander R. . . .Laine M. (2022). The pursuit of effective working memory training: A pre-registered randomised controlled trial with a novel varied training protocol. Journal of Cognitive Enhancement, 6, 232–247.

[bibr36-17470218241278272] Rosseel Y. (2012). Lavaan: An R package for structural equation modeling. Journal of Statistical Software, 48(2), 1–36. 10.18637/jss.v048.i02

[bibr37-17470218241278272] R Studio Team. (2021). RStudio: Integrated development environment for R. Boston, MA. http://www.rstudio.com/

[bibr38-17470218241278272] Sala G. Gobet F. (2017). Working memory training in typically developing children: A meta-analysis of the available evidence. Developmental Psychology, 53(4), 671–685. 10.1037/dev0000265 28165253

[bibr39-17470218241278272] Salmi J. Nyberg L. Laine M. (2018). Working memory training mostly engages general-purpose large-scale networks for learning. Neuroscience and Biobehavioral Reviews, 93, 108–122. 10.1016/j.neubiorev.2018.03.019 29574197

[bibr40-17470218241278272] Schneider W. Chein J. M. (2003). Controlled & automatic processing: Behavior, theory, and biological mechanisms. Cognitive Science, 27(3), 525–559. 10.1207/s15516709cog2703_8

[bibr41-17470218241278272] Schneider W. Shiffrin R. M. (1977). Controlled and automatic human information processing: I. Detection, search, and attention. Psychological Review, 84(1), 1–66. 10.1037/0033-295X.84.1.1

[bibr42-17470218241278272] Schwaighofer M. Fischer F. Bühner M. (2015). Does working memory training transfer? A meta-analysis including training conditions as moderators. Educational Psychologist, 50(2), 138–166. 10.1080/00461520.2015.1036274

[bibr43-17470218241278272] Shiffrin R. M. Schneider W. (1977). Controlled and automatic human information processing: II. Perceptual learning, automatic attending and a general theory. Psychological Review, 84(2), 127–190. 10.1037/0033-295X.84.2.127

[bibr44-17470218241278272] Soveri A. Antfolk J. Karlsson L. Salo B. Laine M. (2017). Working memory training revisited: A multi-level meta-analysis of n-back training studies. Psychonomic Bulletin & Review, 24, 1077–1096. 10.3758/s13423-016-1217-0 28116702

[bibr45-17470218241278272] Szmalec A. Verbruggen F. Vandierendonck A. Kemps E. (2011). Control of interference during working memory updating. Journal of Experimental Psychology: Human Perception and Performance, 37(1), 137–151. 10.1037/a0020365 20731517

[bibr46-17470218241278272] Taatgen N. A. (2013). The nature and transfer of cognitive skills. Psychological Review, 120(3), 439–471. 10.1037/a0033138 23750831

[bibr47-17470218241278272] Waris O. Fellman D. Jylkkä J. Laine M. (2021). Stimulus novelty, task demands, and strategy use in episodic memory. Quarterly Journal of Experimental Psychology, 74(5), 872–888. 10.1177/1747021820980301 PMC8054168 33245018

[bibr48-17470218241278272] Waris O. Jylkkä J. Fellman D. Laine M. (2021). Spontaneous strategy use during a working memory updating task. Acta Psychologica, 212, 103211. 10.1016/j.actpsy.2020.103211 33220613

[bibr49-17470218241278272] Weicker J. Villringer A. Thöne-Otto A. (2016). Can impaired working memory functioning be improved by training? A meta-analysis with a special focus on brain injured patients. Neuropsychology, 30(2), 190–212. 10.1037/neu0000227 26237626

