# Supplementary material for: From task-general towards task-specific cognitive operations in a few minutes? Working memory performance as an adaptive process
Source: Q J Exp Psychol (Hove). 2024 Sep 18;78(8):1547–63. doi: 10.1177/17470218241278272 (PMC12267864; doi:10.1177/17470218241278272)
Supplement: sj-docx-1-qjp-10.1177_17470218241278272 – Supplemental material for From task-general towards task-specific cognitive operations in a few minutes? Working memory performance as an adaptive process [file sj-docx-1-qjp-10.1177_17470218241278272.docx]

| **Table A1.** Descriptive statistics for performances in each task phase in Experiment 1 | | | | | | | |
| --- | --- | --- | --- | --- | --- | --- | --- |
|  | **N** | **M** | **SD** | **Skewness** | **SE of Skewness** | **Kurtosis** | **SE of Kurtosis** |
| **Task phase 1** | | | | | | | |
| BSB | 295 | 0.673 | 0.318 | -0.64 | 0.142 | -0.591 | 0.283 |
| BSD | 295 | 0.664 | 0.35 | -0.433 | 0.142 | -1.323 | 0.283 |
| FSB | 295 | 0.681 | 0.34 | -0.55 | 0.142 | -1.101 | 0.283 |
| FSD | 295 | 0.75 | 0.327 | -1.021 | 0.142 | -0.336 | 0.283 |
| NBB | 294 | 1.575 | 0.36 | -0.167 | 0.142 | -1.301 | 0.283 |
| NBC | 291 | 1.679 | 0.324 | -0.506 | 0.143 | -0.986 | 0.285 |
| NBD | 295 | 1.636 | 0.346 | -0.386 | 0.142 | -1.147 | 0.283 |
| NBL | 293 | 1.636 | 0.339 | -0.344 | 0.142 | -1.159 | 0.284 |
| RMB | 296 | 2.02 | 1.468 | 0.081 | 0.142 | -1.384 | 0.282 |
| RMD | 296 | 2.753 | 1.492 | -0.711 | 0.142 | -1.053 | 0.282 |
| **Task phase 2** | | | | | | | |
| BSB | 295 | 0.678 | 0.269 | -0.748 | 0.142 | -0.145 | 0.283 |
| BSD | 295 | 0.678 | 0.257 | -0.524 | 0.142 | -0.485 | 0.283 |
| FSB | 295 | 0.638 | 0.283 | -0.434 | 0.142 | -0.706 | 0.283 |
| FSD | 295 | 0.684 | 0.262 | -0.602 | 0.142 | -0.334 | 0.283 |
| NBB | 294 | 2.45 | 0.915 | 0.435 | 0.142 | -0.346 | 0.283 |
| NBC | 291 | 2.542 | 0.822 | 0.583 | 0.143 | 0.286 | 0.285 |
| NBD | 295 | 2.584 | 0.933 | 0.368 | 0.142 | -0.283 | 0.283 |
| NBL | 293 | 2.456 | 0.812 | 0.545 | 0.142 | 0.299 | 0.284 |
| RMB | 296 | 2.101 | 1.249 | -0.125 | 0.142 | -1.134 | 0.282 |
| RMD | 296 | 2.762 | 1.143 | -0.603 | 0.142 | -0.712 | 0.282 |
| **Task phase 3** | | | | | | | |
| BSB | 295 | 0.667 | 0.261 | -0.657 | 0.142 | -0.247 | 0.283 |
| BSD | 295 | 0.645 | 0.283 | -0.354 | 0.142 | -0.898 | 0.283 |
| FSB | 295 | 0.657 | 0.289 | -0.597 | 0.142 | -0.457 | 0.283 |
| FSD | 295 | 0.712 | 0.25 | -0.7 | 0.142 | -0.146 | 0.283 |
| NBB | 292 | 2.621 | 1.036 | 0.565 | 0.143 | -0.002 | 0.284 |
| NBC | 290 | 2.722 | 1.069 | 0.869 | 0.143 | 0.657 | 0.285 |
| NBD | 294 | 2.834 | 1.153 | 0.755 | 0.142 | 0.454 | 0.283 |
| NBL | 292 | 2.561 | 1.004 | 0.751 | 0.143 | 0.323 | 0.284 |
| RMB | 296 | 2.216 | 1.266 | -0.199 | 0.142 | -1.077 | 0.282 |
| RMD | 296 | 2.914 | 1.072 | -0.846 | 0.142 | -0.082 | 0.282 |
| **Task phase 4** | | | | | | | |
| BSB | 295 | 0.652 | 0.262 | -0.727 | 0.142 | -0.023 | 0.283 |
| BSD | 295 | 0.642 | 0.274 | -0.366 | 0.142 | -0.802 | 0.283 |
| FSB | 295 | 0.674 | 0.272 | -0.698 | 0.142 | -0.184 | 0.283 |
| FSD | 295 | 0.685 | 0.271 | -0.627 | 0.142 | -0.464 | 0.283 |
| NBB | 293 | 2.68 | 1.095 | 0.688 | 0.142 | 0.23 | 0.284 |
| NBC | 290 | 2.852 | 1.14 | 0.917 | 0.143 | 0.86 | 0.285 |
| NBD | 292 | 2.862 | 1.261 | 0.901 | 0.143 | 0.73 | 0.284 |
| NBL | 291 | 2.711 | 1.154 | 1.011 | 0.143 | 1.032 | 0.285 |
| RMB | 296 | 2.166 | 1.235 | -0.15 | 0.142 | -1.003 | 0.282 |
| RMD | 296 | 2.88 | 1.11 | -0.864 | 0.142 | -0.105 | 0.282 |
| BSB = Backward span with boxes; BSD = Backward span with digits; FSB = Forward span with boxes; FSD = Forward span with digits; NBB = n-back with boxes; NBC = n-back with colors; NBD = N-back with digits; NBL = N-back with letters; RMB = Running memory with boxes; RMD = Running memory with digits. | | | | | | | |

**Table A2.** Intercorrelations between the task performances across the four task phases in Experiment 1. The number after the abbreviation (e.g., BSB1) indicates the task phase. BSB = Backward span with boxes; BSD = Backward span with digits; FSB = Forward span with boxes; FSD = Forward span with digits; NBB = n-back with boxes; NBC = n-back with colors; NBD = N-back with digits; NBL = N-back with letters; RMB = Running memory with boxes; RMD = Running memory with digits. (Note: please copy and paste to another program such as Excel to ease viewing. The data is available also at <https://osf.io/gvqhu/>)

| **Pearson's Correlations** | | | | | | | | | | | | | | | | | | | | | | | | | | | | | | | | | | | | | | | | | |
| --- | --- | --- | --- | --- | --- | --- | --- | --- | --- | --- | --- | --- | --- | --- | --- | --- | --- | --- | --- | --- | --- | --- | --- | --- | --- | --- | --- | --- | --- | --- | --- | --- | --- | --- | --- | --- | --- | --- | --- | --- | --- |
| **Variable** |  | **BSB1** | **BSB2** | **BSB3** | **BSB4** | **BSD1** | **BSD2** | **BSD3** | **BSD4** | **FSB1** | **FSB2** | **FSB3** | **FSB4** | **FSD1** | **FSD2** | **FSD3** | **FSD4** | **NBB1** | **NBB2** | **NBB3** | **NBB4** | **NBC1** | **NBC2** | **NBC3** | **NBC4** | **NBD1** | **NBD2** | **NBD3** | **NBD4** | **NBL1** | **NBL2** | **NBL3** | **NBL4** | **RMB1** | **RMB2** | **RMB3** | **RMB4** | **RMD1** | **RMD2** | **RMD3** | **RMD4** |
| 1. BSB1 | Pearson's r | — |  |  |  |  |  |  |  |  |  |  |  |  |  |  |  |  |  |  |  |  |  |  |  |  |  |  |  |  |  |  |  |  |  |  |  |  |  |  |  |
|  | p-value | — |  |  |  |  |  |  |  |  |  |  |  |  |  |  |  |  |  |  |  |  |  |  |  |  |  |  |  |  |  |  |  |  |  |  |  |  |  |  |  |
| 2. BSB2 | Pearson's r | 0.407 | — |  |  |  |  |  |  |  |  |  |  |  |  |  |  |  |  |  |  |  |  |  |  |  |  |  |  |  |  |  |  |  |  |  |  |  |  |  |  |
|  | p-value | < .001 | — |  |  |  |  |  |  |  |  |  |  |  |  |  |  |  |  |  |  |  |  |  |  |  |  |  |  |  |  |  |  |  |  |  |  |  |  |  |  |
| 3. BSB3 | Pearson's r | 0.418 | 0.506 | — |  |  |  |  |  |  |  |  |  |  |  |  |  |  |  |  |  |  |  |  |  |  |  |  |  |  |  |  |  |  |  |  |  |  |  |  |  |
|  | p-value | < .001 | < .001 | — |  |  |  |  |  |  |  |  |  |  |  |  |  |  |  |  |  |  |  |  |  |  |  |  |  |  |  |  |  |  |  |  |  |  |  |  |  |
| 4. BSB4 | Pearson's r | 0.4 | 0.488 | 0.481 | — |  |  |  |  |  |  |  |  |  |  |  |  |  |  |  |  |  |  |  |  |  |  |  |  |  |  |  |  |  |  |  |  |  |  |  |  |
|  | p-value | < .001 | < .001 | < .001 | — |  |  |  |  |  |  |  |  |  |  |  |  |  |  |  |  |  |  |  |  |  |  |  |  |  |  |  |  |  |  |  |  |  |  |  |  |
| 5. BSD1 | Pearson's r | 0.177 | 0.258 | 0.265 | 0.288 | — |  |  |  |  |  |  |  |  |  |  |  |  |  |  |  |  |  |  |  |  |  |  |  |  |  |  |  |  |  |  |  |  |  |  |  |
|  | p-value | 0.002 | < .001 | < .001 | < .001 | — |  |  |  |  |  |  |  |  |  |  |  |  |  |  |  |  |  |  |  |  |  |  |  |  |  |  |  |  |  |  |  |  |  |  |  |
| 6. BSD2 | Pearson's r | 0.254 | 0.218 | 0.207 | 0.255 | 0.379 | — |  |  |  |  |  |  |  |  |  |  |  |  |  |  |  |  |  |  |  |  |  |  |  |  |  |  |  |  |  |  |  |  |  |  |
|  | p-value | < .001 | < .001 | < .001 | < .001 | < .001 | — |  |  |  |  |  |  |  |  |  |  |  |  |  |  |  |  |  |  |  |  |  |  |  |  |  |  |  |  |  |  |  |  |  |  |
| 7. BSD3 | Pearson's r | 0.247 | 0.315 | 0.34 | 0.308 | 0.275 | 0.392 | — |  |  |  |  |  |  |  |  |  |  |  |  |  |  |  |  |  |  |  |  |  |  |  |  |  |  |  |  |  |  |  |  |  |
|  | p-value | < .001 | < .001 | < .001 | < .001 | < .001 | < .001 | — |  |  |  |  |  |  |  |  |  |  |  |  |  |  |  |  |  |  |  |  |  |  |  |  |  |  |  |  |  |  |  |  |  |
| 8. BSD4 | Pearson's r | 0.194 | 0.298 | 0.331 | 0.287 | 0.284 | 0.365 | 0.469 | — |  |  |  |  |  |  |  |  |  |  |  |  |  |  |  |  |  |  |  |  |  |  |  |  |  |  |  |  |  |  |  |  |
|  | p-value | < .001 | < .001 | < .001 | < .001 | < .001 | < .001 | < .001 | — |  |  |  |  |  |  |  |  |  |  |  |  |  |  |  |  |  |  |  |  |  |  |  |  |  |  |  |  |  |  |  |  |
| 9. FSB1 | Pearson's r | 0.098 | 0.209 | 0.19 | 0.141 | 0.007 | 0.082 | 0.034 | 0.025 | — |  |  |  |  |  |  |  |  |  |  |  |  |  |  |  |  |  |  |  |  |  |  |  |  |  |  |  |  |  |  |  |
|  | p-value | 0.094 | < .001 | 0.001 | 0.015 | 0.91 | 0.161 | 0.558 | 0.673 | — |  |  |  |  |  |  |  |  |  |  |  |  |  |  |  |  |  |  |  |  |  |  |  |  |  |  |  |  |  |  |  |
| 10. FSB2 | Pearson's r | 0.206 | 0.256 | 0.259 | 0.284 | 0.032 | 0.153 | 0.204 | 0.141 | 0.302 | — |  |  |  |  |  |  |  |  |  |  |  |  |  |  |  |  |  |  |  |  |  |  |  |  |  |  |  |  |  |  |
|  | p-value | < .001 | < .001 | < .001 | < .001 | 0.582 | 0.008 | < .001 | 0.016 | < .001 | — |  |  |  |  |  |  |  |  |  |  |  |  |  |  |  |  |  |  |  |  |  |  |  |  |  |  |  |  |  |  |
| 11. FSB3 | Pearson's r | 0.247 | 0.34 | 0.33 | 0.315 | 0.23 | 0.147 | 0.246 | 0.282 | 0.191 | 0.216 | — |  |  |  |  |  |  |  |  |  |  |  |  |  |  |  |  |  |  |  |  |  |  |  |  |  |  |  |  |  |
|  | p-value | < .001 | < .001 | < .001 | < .001 | < .001 | 0.012 | < .001 | < .001 | < .001 | < .001 | — |  |  |  |  |  |  |  |  |  |  |  |  |  |  |  |  |  |  |  |  |  |  |  |  |  |  |  |  |  |
| 12. FSB4 | Pearson's r | 0.306 | 0.304 | 0.238 | 0.303 | 0.149 | 0.304 | 0.295 | 0.194 | 0.268 | 0.287 | 0.327 | — |  |  |  |  |  |  |  |  |  |  |  |  |  |  |  |  |  |  |  |  |  |  |  |  |  |  |  |  |
|  | p-value | < .001 | < .001 | < .001 | < .001 | 0.011 | < .001 | < .001 | < .001 | < .001 | < .001 | < .001 | — |  |  |  |  |  |  |  |  |  |  |  |  |  |  |  |  |  |  |  |  |  |  |  |  |  |  |  |  |
| 13. FSD1 | Pearson's r | 0.07 | 0.081 | 0.146 | 0.121 | 0.184 | 0.168 | 0.206 | 0.159 | 0.016 | 0.073 | 0.103 | 0.168 | — |  |  |  |  |  |  |  |  |  |  |  |  |  |  |  |  |  |  |  |  |  |  |  |  |  |  |  |
|  | p-value | 0.233 | 0.166 | 0.012 | 0.038 | 0.002 | 0.004 | < .001 | 0.006 | 0.787 | 0.214 | 0.077 | 0.004 | — |  |  |  |  |  |  |  |  |  |  |  |  |  |  |  |  |  |  |  |  |  |  |  |  |  |  |  |
| 14. FSD2 | Pearson's r | 0.12 | 0.175 | 0.143 | 0.162 | 0.27 | 0.367 | 0.407 | 0.272 | 0.125 | 0.164 | 0.223 | 0.261 | 0.24 | — |  |  |  |  |  |  |  |  |  |  |  |  |  |  |  |  |  |  |  |  |  |  |  |  |  |  |
|  | p-value | 0.039 | 0.002 | 0.014 | 0.005 | < .001 | < .001 | < .001 | < .001 | 0.032 | 0.005 | < .001 | < .001 | < .001 | — |  |  |  |  |  |  |  |  |  |  |  |  |  |  |  |  |  |  |  |  |  |  |  |  |  |  |
| 15. FSD3 | Pearson's r | 0.139 | 0.195 | 0.164 | 0.178 | 0.21 | 0.345 | 0.331 | 0.34 | 0.066 | 0.12 | 0.184 | 0.258 | 0.143 | 0.315 | — |  |  |  |  |  |  |  |  |  |  |  |  |  |  |  |  |  |  |  |  |  |  |  |  |  |
|  | p-value | 0.017 | < .001 | 0.005 | 0.002 | < .001 | < .001 | < .001 | < .001 | 0.262 | 0.04 | 0.001 | < .001 | 0.014 | < .001 | — |  |  |  |  |  |  |  |  |  |  |  |  |  |  |  |  |  |  |  |  |  |  |  |  |  |
| 16. FSD4 | Pearson's r | 0.192 | 0.183 | 0.226 | 0.235 | 0.225 | 0.346 | 0.343 | 0.29 | 0.096 | 0.166 | 0.221 | 0.303 | 0.131 | 0.26 | 0.242 | — |  |  |  |  |  |  |  |  |  |  |  |  |  |  |  |  |  |  |  |  |  |  |  |  |
|  | p-value | < .001 | 0.002 | < .001 | < .001 | < .001 | < .001 | < .001 | < .001 | 0.099 | 0.004 | < .001 | < .001 | 0.024 | < .001 | < .001 | — |  |  |  |  |  |  |  |  |  |  |  |  |  |  |  |  |  |  |  |  |  |  |  |  |
| 17. NBB1 | Pearson's r | 0.1 | 0.204 | 0.234 | 0.318 | 0.201 | 0.195 | 0.24 | 0.165 | 0.155 | 0.164 | 0.164 | 0.214 | -0.002 | 0.095 | 0.135 | 0.154 | — |  |  |  |  |  |  |  |  |  |  |  |  |  |  |  |  |  |  |  |  |  |  |  |
|  | p-value | 0.087 | < .001 | < .001 | < .001 | < .001 | < .001 | < .001 | 0.005 | 0.008 | 0.005 | 0.005 | < .001 | 0.976 | 0.104 | 0.02 | 0.008 | — |  |  |  |  |  |  |  |  |  |  |  |  |  |  |  |  |  |  |  |  |  |  |  |
| 18. NBB2 | Pearson's r | 0.231 | 0.304 | 0.331 | 0.401 | 0.303 | 0.323 | 0.335 | 0.309 | 0.175 | 0.28 | 0.29 | 0.251 | 0.056 | 0.194 | 0.187 | 0.156 | 0.599 | — |  |  |  |  |  |  |  |  |  |  |  |  |  |  |  |  |  |  |  |  |  |  |
|  | p-value | < .001 | < .001 | < .001 | < .001 | < .001 | < .001 | < .001 | < .001 | 0.003 | < .001 | < .001 | < .001 | 0.339 | < .001 | 0.001 | 0.007 | < .001 | — |  |  |  |  |  |  |  |  |  |  |  |  |  |  |  |  |  |  |  |  |  |  |
| 19. NBB3 | Pearson's r | 0.236 | 0.288 | 0.277 | 0.338 | 0.237 | 0.304 | 0.358 | 0.327 | 0.149 | 0.26 | 0.179 | 0.244 | 0.106 | 0.208 | 0.221 | 0.192 | 0.521 | 0.776 | — |  |  |  |  |  |  |  |  |  |  |  |  |  |  |  |  |  |  |  |  |  |
|  | p-value | < .001 | < .001 | < .001 | < .001 | < .001 | < .001 | < .001 | < .001 | 0.011 | < .001 | 0.002 | < .001 | 0.071 | < .001 | < .001 | < .001 | < .001 | < .001 | — |  |  |  |  |  |  |  |  |  |  |  |  |  |  |  |  |  |  |  |  |  |
| 20. NBB4 | Pearson's r | 0.258 | 0.276 | 0.256 | 0.33 | 0.181 | 0.282 | 0.323 | 0.331 | 0.167 | 0.295 | 0.274 | 0.237 | 0.119 | 0.122 | 0.141 | 0.162 | 0.481 | 0.675 | 0.805 | — |  |  |  |  |  |  |  |  |  |  |  |  |  |  |  |  |  |  |  |  |
|  | p-value | < .001 | < .001 | < .001 | < .001 | 0.002 | < .001 | < .001 | < .001 | 0.004 | < .001 | < .001 | < .001 | 0.041 | 0.037 | 0.016 | 0.005 | < .001 | < .001 | < .001 | — |  |  |  |  |  |  |  |  |  |  |  |  |  |  |  |  |  |  |  |  |
| 21. NBC1 | Pearson's r | 0.264 | 0.248 | 0.214 | 0.218 | 0.119 | 0.106 | 0.132 | 0.19 | 0.09 | 0.195 | 0.241 | 0.175 | 0.08 | 0.049 | 0.14 | 0.16 | 0.169 | 0.268 | 0.305 | 0.368 | — |  |  |  |  |  |  |  |  |  |  |  |  |  |  |  |  |  |  |  |
|  | p-value | < .001 | < .001 | < .001 | < .001 | 0.043 | 0.072 | 0.025 | 0.001 | 0.126 | < .001 | < .001 | 0.003 | 0.172 | 0.401 | 0.017 | 0.006 | 0.004 | < .001 | < .001 | < .001 | — |  |  |  |  |  |  |  |  |  |  |  |  |  |  |  |  |  |  |  |
| 22. NBC2 | Pearson's r | 0.203 | 0.358 | 0.337 | 0.322 | 0.258 | 0.206 | 0.229 | 0.277 | 0.1 | 0.191 | 0.287 | 0.188 | 0.101 | 0.057 | 0.178 | 0.137 | 0.273 | 0.464 | 0.555 | 0.544 | 0.539 | — |  |  |  |  |  |  |  |  |  |  |  |  |  |  |  |  |  |  |
|  | p-value | < .001 | < .001 | < .001 | < .001 | < .001 | < .001 | < .001 | < .001 | 0.089 | 0.001 | < .001 | 0.001 | 0.085 | 0.332 | 0.002 | 0.019 | < .001 | < .001 | < .001 | < .001 | < .001 | — |  |  |  |  |  |  |  |  |  |  |  |  |  |  |  |  |  |  |
| 23. NBC3 | Pearson's r | 0.202 | 0.295 | 0.313 | 0.278 | 0.234 | 0.241 | 0.291 | 0.294 | 0.14 | 0.258 | 0.28 | 0.221 | 0.101 | 0.097 | 0.202 | 0.15 | 0.317 | 0.478 | 0.54 | 0.537 | 0.434 | 0.774 | — |  |  |  |  |  |  |  |  |  |  |  |  |  |  |  |  |  |
|  | p-value | < .001 | < .001 | < .001 | < .001 | < .001 | < .001 | < .001 | < .001 | 0.017 | < .001 | < .001 | < .001 | 0.087 | 0.1 | < .001 | 0.011 | < .001 | < .001 | < .001 | < .001 | < .001 | < .001 | — |  |  |  |  |  |  |  |  |  |  |  |  |  |  |  |  |  |
| 24. NBC4 | Pearson's r | 0.217 | 0.301 | 0.279 | 0.317 | 0.231 | 0.247 | 0.346 | 0.311 | 0.154 | 0.249 | 0.314 | 0.229 | 0.082 | 0.099 | 0.211 | 0.174 | 0.328 | 0.506 | 0.579 | 0.598 | 0.403 | 0.677 | 0.822 | — |  |  |  |  |  |  |  |  |  |  |  |  |  |  |  |  |
|  | p-value | < .001 | < .001 | < .001 | < .001 | < .001 | < .001 | < .001 | < .001 | 0.009 | < .001 | < .001 | < .001 | 0.162 | 0.091 | < .001 | 0.003 | < .001 | < .001 | < .001 | < .001 | < .001 | < .001 | < .001 | — |  |  |  |  |  |  |  |  |  |  |  |  |  |  |  |  |
| 25. NBD1 | Pearson's r | 0.168 | 0.248 | 0.24 | 0.252 | 0.186 | 0.174 | 0.222 | 0.195 | 0.091 | 0.174 | 0.177 | 0.153 | 0.078 | 0.029 | 0.057 | 0.072 | 0.25 | 0.285 | 0.321 | 0.306 | 0.115 | 0.296 | 0.334 | 0.367 | — |  |  |  |  |  |  |  |  |  |  |  |  |  |  |  |
|  | p-value | 0.004 | < .001 | < .001 | < .001 | 0.001 | 0.003 | < .001 | < .001 | 0.121 | 0.003 | 0.002 | 0.009 | 0.18 | 0.622 | 0.326 | 0.218 | < .001 | < .001 | < .001 | < .001 | 0.051 | < .001 | < .001 | < .001 | — |  |  |  |  |  |  |  |  |  |  |  |  |  |  |  |
| 26. NBD2 | Pearson's r | 0.235 | 0.334 | 0.284 | 0.393 | 0.236 | 0.299 | 0.376 | 0.265 | 0.159 | 0.293 | 0.242 | 0.279 | 0.188 | 0.14 | 0.186 | 0.233 | 0.402 | 0.537 | 0.591 | 0.543 | 0.275 | 0.486 | 0.52 | 0.568 | 0.568 | — |  |  |  |  |  |  |  |  |  |  |  |  |  |  |
|  | p-value | < .001 | < .001 | < .001 | < .001 | < .001 | < .001 | < .001 | < .001 | 0.006 | < .001 | < .001 | < .001 | 0.001 | 0.016 | 0.001 | < .001 | < .001 | < .001 | < .001 | < .001 | < .001 | < .001 | < .001 | < .001 | < .001 | — |  |  |  |  |  |  |  |  |  |  |  |  |  |  |
| 27. NBD3 | Pearson's r | 0.23 | 0.304 | 0.318 | 0.377 | 0.236 | 0.303 | 0.357 | 0.278 | 0.149 | 0.24 | 0.238 | 0.269 | 0.137 | 0.189 | 0.231 | 0.167 | 0.402 | 0.527 | 0.576 | 0.556 | 0.269 | 0.55 | 0.609 | 0.652 | 0.509 | 0.789 | — |  |  |  |  |  |  |  |  |  |  |  |  |  |
|  | p-value | < .001 | < .001 | < .001 | < .001 | < .001 | < .001 | < .001 | < .001 | 0.011 | < .001 | < .001 | < .001 | 0.019 | 0.001 | < .001 | 0.004 | < .001 | < .001 | < .001 | < .001 | < .001 | < .001 | < .001 | < .001 | < .001 | < .001 | — |  |  |  |  |  |  |  |  |  |  |  |  |  |
| 28. NBD4 | Pearson's r | 0.2 | 0.321 | 0.287 | 0.368 | 0.242 | 0.242 | 0.314 | 0.256 | 0.12 | 0.264 | 0.252 | 0.252 | 0.125 | 0.138 | 0.21 | 0.164 | 0.384 | 0.539 | 0.595 | 0.573 | 0.282 | 0.563 | 0.652 | 0.702 | 0.457 | 0.689 | 0.843 | — |  |  |  |  |  |  |  |  |  |  |  |  |
|  | p-value | < .001 | < .001 | < .001 | < .001 | < .001 | < .001 | < .001 | < .001 | 0.041 | < .001 | < .001 | < .001 | 0.033 | 0.018 | < .001 | 0.005 | < .001 | < .001 | < .001 | < .001 | < .001 | < .001 | < .001 | < .001 | < .001 | < .001 | < .001 | — |  |  |  |  |  |  |  |  |  |  |  |  |
| 29. NBL1 | Pearson's r | 0.229 | 0.216 | 0.233 | 0.339 | 0.199 | 0.203 | 0.153 | 0.195 | 0.107 | 0.24 | 0.27 | 0.291 | 0.155 | 0.131 | 0.143 | 0.167 | 0.235 | 0.334 | 0.3 | 0.321 | 0.111 | 0.256 | 0.326 | 0.3 | 0.225 | 0.335 | 0.315 | 0.316 | — |  |  |  |  |  |  |  |  |  |  |  |
|  | p-value | < .001 | < .001 | < .001 | < .001 | < .001 | < .001 | 0.009 | < .001 | 0.068 | < .001 | < .001 | < .001 | 0.008 | 0.025 | 0.014 | 0.004 | < .001 | < .001 | < .001 | < .001 | 0.059 | < .001 | < .001 | < .001 | < .001 | < .001 | < .001 | < .001 | — |  |  |  |  |  |  |  |  |  |  |  |
| 30. NBL2 | Pearson's r | 0.272 | 0.267 | 0.309 | 0.383 | 0.266 | 0.207 | 0.244 | 0.295 | 0.013 | 0.172 | 0.264 | 0.243 | 0.157 | 0.159 | 0.159 | 0.192 | 0.278 | 0.448 | 0.488 | 0.54 | 0.274 | 0.458 | 0.511 | 0.532 | 0.34 | 0.531 | 0.578 | 0.54 | 0.533 | — |  |  |  |  |  |  |  |  |  |  |
|  | p-value | < .001 | < .001 | < .001 | < .001 | < .001 | < .001 | < .001 | < .001 | 0.82 | 0.003 | < .001 | < .001 | 0.007 | 0.007 | 0.006 | < .001 | < .001 | < .001 | < .001 | < .001 | < .001 | < .001 | < .001 | < .001 | < .001 | < .001 | < .001 | < .001 | < .001 | — |  |  |  |  |  |  |  |  |  |  |
| 31. NBL3 | Pearson's r | 0.228 | 0.276 | 0.291 | 0.345 | 0.213 | 0.243 | 0.271 | 0.321 | 0.085 | 0.159 | 0.231 | 0.199 | 0.076 | 0.137 | 0.166 | 0.185 | 0.308 | 0.517 | 0.557 | 0.578 | 0.341 | 0.522 | 0.613 | 0.624 | 0.371 | 0.616 | 0.625 | 0.621 | 0.389 | 0.738 | — |  |  |  |  |  |  |  |  |  |
|  | p-value | < .001 | < .001 | < .001 | < .001 | < .001 | < .001 | < .001 | < .001 | 0.148 | 0.007 | < .001 | < .001 | 0.196 | 0.019 | 0.004 | 0.001 | < .001 | < .001 | < .001 | < .001 | < .001 | < .001 | < .001 | < .001 | < .001 | < .001 | < .001 | < .001 | < .001 | < .001 | — |  |  |  |  |  |  |  |  |  |
| 32. NBL4 | Pearson's r | 0.232 | 0.324 | 0.298 | 0.38 | 0.222 | 0.319 | 0.319 | 0.292 | 0.154 | 0.228 | 0.255 | 0.219 | 0.101 | 0.185 | 0.244 | 0.206 | 0.325 | 0.506 | 0.556 | 0.576 | 0.331 | 0.522 | 0.584 | 0.633 | 0.346 | 0.608 | 0.656 | 0.667 | 0.328 | 0.627 | 0.791 | — |  |  |  |  |  |  |  |  |
|  | p-value | < .001 | < .001 | < .001 | < .001 | < .001 | < .001 | < .001 | < .001 | 0.008 | < .001 | < .001 | < .001 | 0.085 | 0.002 | < .001 | < .001 | < .001 | < .001 | < .001 | < .001 | < .001 | < .001 | < .001 | < .001 | < .001 | < .001 | < .001 | < .001 | < .001 | < .001 | < .001 | — |  |  |  |  |  |  |  |  |
| 33. RMB1 | Pearson's r | 0.15 | 0.164 | 0.226 | 0.264 | 0.151 | 0.226 | 0.19 | 0.179 | 0.054 | 0.212 | 0.173 | 0.234 | 0.174 | 0.051 | 0.077 | 0.137 | 0.159 | 0.193 | 0.225 | 0.261 | 0.207 | 0.259 | 0.241 | 0.259 | 0.262 | 0.298 | 0.207 | 0.257 | 0.177 | 0.254 | 0.245 | 0.221 | — |  |  |  |  |  |  |  |
|  | p-value | 0.01 | 0.005 | < .001 | < .001 | 0.009 | < .001 | 0.001 | 0.002 | 0.358 | < .001 | 0.003 | < .001 | 0.003 | 0.386 | 0.19 | 0.019 | 0.006 | < .001 | < .001 | < .001 | < .001 | < .001 | < .001 | < .001 | < .001 | < .001 | < .001 | < .001 | 0.002 | < .001 | < .001 | < .001 | — |  |  |  |  |  |  |  |
| 34. RMB2 | Pearson's r | 0.175 | 0.272 | 0.364 | 0.244 | 0.208 | 0.222 | 0.21 | 0.184 | 0.086 | 0.135 | 0.251 | 0.226 | 0.107 | 0.173 | 0.113 | 0.214 | 0.23 | 0.246 | 0.271 | 0.205 | 0.123 | 0.237 | 0.207 | 0.209 | 0.192 | 0.28 | 0.265 | 0.255 | 0.148 | 0.177 | 0.269 | 0.27 | 0.347 | — |  |  |  |  |  |  |
|  | p-value | 0.003 | < .001 | < .001 | < .001 | < .001 | < .001 | < .001 | 0.001 | 0.139 | 0.021 | < .001 | < .001 | 0.066 | 0.003 | 0.054 | < .001 | < .001 | < .001 | < .001 | < .001 | 0.035 | < .001 | < .001 | < .001 | < .001 | < .001 | < .001 | < .001 | 0.011 | 0.002 | < .001 | < .001 | < .001 | — |  |  |  |  |  |  |
| 35. RMB3 | Pearson's r | 0.197 | 0.258 | 0.354 | 0.29 | 0.225 | 0.201 | 0.266 | 0.195 | 0.112 | 0.13 | 0.211 | 0.176 | 0.09 | 0.137 | 0.106 | 0.162 | 0.179 | 0.257 | 0.271 | 0.256 | 0.253 | 0.251 | 0.237 | 0.296 | 0.163 | 0.323 | 0.322 | 0.277 | 0.138 | 0.224 | 0.334 | 0.338 | 0.265 | 0.525 | — |  |  |  |  |  |
|  | p-value | < .001 | < .001 | < .001 | < .001 | < .001 | < .001 | < .001 | < .001 | 0.054 | 0.026 | < .001 | 0.002 | 0.123 | 0.019 | 0.07 | 0.005 | 0.002 | < .001 | < .001 | < .001 | < .001 | < .001 | < .001 | < .001 | 0.005 | < .001 | < .001 | < .001 | 0.018 | < .001 | < .001 | < .001 | < .001 | < .001 | — |  |  |  |  |  |
| 36. RMB4 | Pearson's r | 0.191 | 0.244 | 0.288 | 0.239 | 0.152 | 0.15 | 0.165 | 0.152 | 0.076 | 0.162 | 0.213 | 0.133 | 0.113 | 0.105 | 0.066 | 0.172 | 0.153 | 0.226 | 0.161 | 0.186 | 0.156 | 0.179 | 0.182 | 0.253 | 0.196 | 0.274 | 0.265 | 0.25 | 0.153 | 0.215 | 0.221 | 0.218 | 0.332 | 0.368 | 0.498 | — |  |  |  |  |
|  | p-value | < .001 | < .001 | < .001 | < .001 | 0.009 | 0.01 | 0.004 | 0.009 | 0.195 | 0.005 | < .001 | 0.023 | 0.052 | 0.072 | 0.258 | 0.003 | 0.009 | < .001 | 0.006 | 0.001 | 0.008 | 0.002 | 0.002 | < .001 | < .001 | < .001 | < .001 | < .001 | 0.009 | < .001 | < .001 | < .001 | < .001 | < .001 | < .001 | — |  |  |  |  |
| 37. RMD1 | Pearson's r | 0.043 | 0.128 | 0.168 | 0.16 | 0.174 | 0.154 | 0.124 | 0.216 | 0.039 | 0.073 | 0.212 | 0.058 | 0.067 | 0.142 | 0.173 | 0.181 | 0.088 | 0.188 | 0.181 | 0.091 | 0.109 | 0.16 | 0.173 | 0.14 | 0.11 | 0.165 | 0.157 | 0.198 | 0.045 | 0.146 | 0.186 | 0.188 | 0.142 | 0.208 | 0.166 | 0.133 | — |  |  |  |
|  | p-value | 0.465 | 0.028 | 0.004 | 0.006 | 0.003 | 0.008 | 0.034 | < .001 | 0.51 | 0.212 | < .001 | 0.319 | 0.253 | 0.015 | 0.003 | 0.002 | 0.13 | 0.001 | 0.002 | 0.119 | 0.064 | 0.006 | 0.003 | 0.017 | 0.058 | 0.005 | 0.007 | < .001 | 0.439 | 0.012 | 0.001 | 0.001 | 0.015 | < .001 | 0.004 | 0.023 | — |  |  |  |
| 38. RMD2 | Pearson's r | 0.077 | 0.159 | 0.207 | 0.133 | 0.261 | 0.263 | 0.32 | 0.261 | 0.062 | 0.114 | 0.125 | 0.155 | 0.111 | 0.181 | 0.247 | 0.172 | 0.208 | 0.255 | 0.297 | 0.212 | 0.148 | 0.203 | 0.254 | 0.195 | 0.08 | 0.206 | 0.286 | 0.21 | 0.125 | 0.221 | 0.227 | 0.292 | 0.087 | 0.13 | 0.197 | 0.141 | 0.246 | — |  |  |
|  | p-value | 0.19 | 0.006 | < .001 | 0.022 | < .001 | < .001 | < .001 | < .001 | 0.287 | 0.051 | 0.032 | 0.007 | 0.057 | 0.002 | < .001 | 0.003 | < .001 | < .001 | < .001 | < .001 | 0.012 | < .001 | < .001 | < .001 | 0.169 | < .001 | < .001 | < .001 | 0.032 | < .001 | < .001 | < .001 | 0.137 | 0.026 | < .001 | 0.015 | < .001 | — |  |  |
| 39. RMD3 | Pearson's r | 0.111 | 0.182 | 0.158 | 0.193 | 0.119 | 0.196 | 0.234 | 0.283 | 0.093 | 0.198 | 0.114 | 0.195 | 0.048 | 0.146 | 0.206 | 0.179 | 0.076 | 0.192 | 0.201 | 0.119 | 0.228 | 0.204 | 0.259 | 0.23 | 0.156 | 0.267 | 0.281 | 0.252 | 0.073 | 0.18 | 0.19 | 0.179 | 0.246 | 0.212 | 0.205 | 0.196 | 0.333 | 0.291 | — |  |
|  | p-value | 0.057 | 0.002 | 0.006 | < .001 | 0.042 | < .001 | < .001 | < .001 | 0.112 | < .001 | 0.049 | < .001 | 0.416 | 0.012 | < .001 | 0.002 | 0.197 | < .001 | < .001 | 0.041 | < .001 | < .001 | < .001 | < .001 | 0.007 | < .001 | < .001 | < .001 | 0.21 | 0.002 | 0.001 | 0.002 | < .001 | < .001 | < .001 | < .001 | < .001 | < .001 | — |  |
| 40. RMD4 | Pearson's r | 0.114 | 0.203 | 0.155 | 0.123 | 0.219 | 0.354 | 0.213 | 0.225 | -0.026 | 0.109 | 0.168 | 0.165 | 0.085 | 0.247 | 0.166 | 0.167 | 0.147 | 0.193 | 0.188 | 0.138 | 0.086 | 0.152 | 0.225 | 0.172 | 0.128 | 0.184 | 0.223 | 0.2 | 0.15 | 0.185 | 0.215 | 0.221 | 0.167 | 0.26 | 0.172 | 0.19 | 0.323 | 0.283 | 0.382 | — |
|  | p-value | 0.05 | < .001 | 0.008 | 0.035 | < .001 | < .001 | < .001 | < .001 | 0.66 | 0.061 | 0.004 | 0.005 | 0.144 | < .001 | 0.004 | 0.004 | 0.011 | < .001 | 0.001 | 0.018 | 0.143 | 0.009 | < .001 | 0.003 | 0.028 | 0.001 | < .001 | < .001 | 0.01 | 0.001 | < .001 | < .001 | 0.004 | < .001 | 0.003 | 0.001 | < .001 | < .001 | < .001 | — |
|  | | | | | | | | | | | | | | | | | | | | | | | | | | | | | | | | | | | | | | | | | |
