# Supplementary material for: From task-general towards task-specific cognitive operations in a few minutes? Working memory performance as an adaptive process
Source: Q J Exp Psychol (Hove). 2024 Sep 18;78(8):1547–63. doi: 10.1177/17470218241278272 (PMC12267864; doi:10.1177/17470218241278272)
Supplement: sj-docx-2-qjp-10.1177_17470218241278272 – Supplemental material for From task-general towards task-specific cognitive operations in a few minutes? Working memory performance as an adaptive process [file sj-docx-2-qjp-10.1177_17470218241278272.docx]

**Appendix B: Item Loading Tables**

| **B1.** Experiment 1 Item Loadings | | | | | | | | |  |
| --- | --- | --- | --- | --- | --- | --- | --- | --- | --- |
| Variable | Estimate | | Standard  Error | | *p*-value | | Standardized  Estimate | |  |
| NBD_T1 | 1.106 | | 0.021 | | < .001 | | 0.466 | |  |
| NBL_T1 | 0.952 | | 0.021 | | < .001 | | 0.418 | |  |
| NBC_T1 | 0.988 | | 0.021 | | < .001 | | 0.442 | |  |
| NBB_T1 | 0.954 | | 0.022 | | < .001 | | 0.398 | |  |
| NBD_T2 | 1.106 | | 0.021 | | < .001 | | 0.738 | |  |
| NBL_T2 | 0.952 | | 0.021 | | < .001 | | 0.707 | |  |
| NBC_T2 | 0.988 | | 0.021 | | < .001 | | 0.710 | |  |
| NBB_T2 | 0.954 | | 0.022 | | < .001 | | 0.649 | |  |
| NBD_T3 | 1.106 | | 0.021 | | < .001 | | 0.809 | |  |
| NBL_T3 | 0.952 | | 0.021 | | < .001 | | 0.784 | |  |
| NBC_T3 | 0.988 | | 0.021 | | < .001 | | 0.758 | |  |
| NBB_T3 | 0.954 | | 0.022 | | < .001 | | 0.755 | |  |
| NBD_T4 | 1.106 | | 0.021 | | < .001 | | 0.825 | |  |
| NBL_T4 | 0.952 | | 0.021 | | < .001 | | 0.794 | |  |
| NBC_T4 | 0.988 | | 0.021 | | < .001 | | 0.813 | |  |
| NBB_T4 | 0.954 | | 0.022 | | < .001 | | 0.769 | |  |
| RMB_T1 | 1.064 | | 0.068 | | < .001 | | 0.399 | |  |
| RMD_T1 | 0.936 | | 0.068 | | < .001 | | 0.337 | |  |
| RMB_T2 | 1.064 | | 0.068 | | < .001 | | 0.368 | |  |
| RMD_T2 | 0.936 | | 0.068 | | < .001 | | 0.355 | |  |
| RMB_T3 | 1.064 | | 0.068 | | < .001 | | 0.451 | |  |
| RMD_T3 | 0.936 | | 0.068 | | < .001 | | 0.459 | |  |
| RMB_T4 | 1.064 | | 0.068 | | < .001 | | 0.435 | |  |
| RMD_T4 | 0.936 | | 0.068 | | < .001 | | 0.438 | |  |
| FSD_T1 | 0.836 | | 0.064 | | < .001 | | 0.266 | |  |
| FSB_T1 | 0.905 | | 0.069 | | < .001 | | 0.274 | |  |
| BSD_T1 | 1.172 | | 0.067 | | < .001 | | 0.362 | |  |
| BSB_T1 | 1.087 | | 0.072 | | < .001 | | 0.364 | |  |
| FSD_T2 | 0.836 | | 0.064 | | < .001 | | 0.407 | |  |
| FSB_T2 | 0.905 | | 0.069 | | < .001 | | 0.396 | |  |
| BSD_T2 | 1.172 | | 0.067 | | < .001 | | 0.569 | |  |
| BSB_T2 | 1.087 | | 0.072 | | < .001 | | 0.516 | |  |
| FSD_T3 | 0.836 | | 0.064 | | < .001 | | 0.460 | |  |
| FSB_T3 | 0.905 | | 0.069 | | < .001 | | 0.443 | |  |
| BSD_T3 | 1.172 | | 0.067 | | < .001 | | 0.587 | |  |
| BSB_T3 | 1.087 | | 0.072 | | < .001 | | 0.573 | |  |
| FSD_T4 | 0.836 | | 0.064 | | < .001 | | 0.427 | |  |
| FSB_T4 | 0.905 | | 0.069 | | < .001 | | 0.461 | |  |
| BSD_T4 | 1.172 | | 0.067 | | < .001 | | 0.574 | |  |
| BSB_T4 | 1.087 | | 0.072 | | < .001 | | 0.572 | |  |
| Note: Parameter estimates reflect the Strong invariant model. T=task phase. | | | | | | | | |  |
| **B2.** Experiment 2 Item Loadings | | | | | | | | | |
| Variable | | Estimate | | Standard | | *p*-value | | Standardized | |
|  |  |  |  | Error | |  |  | Estimate | |
| NBD_T1 | | 0.228 | | 0.06 | | < .001 | | 0.22 | |
| NBL_T1 | | 1.519 | | 0.059 | | < .001 | | 0.832 | |
| NBC_T1 | | 1.253 | | 0.056 | | < .001 | | 0.775 | |
| NBD_T2 | | 1.192 | | 0.039 | | < .001 | | 0.675 | |
| NBL_T2 | | 1.051 | | 0.037 | | < .001 | | 0.689 | |
| NBC_T2 | | 0.756 | | 0.041 | | < .001 | | 0.523 | |
| NBD_T3 | | 1.192 | | 0.039 | | < .001 | | 0.791 | |
| NBL_T3 | | 1.051 | | 0.037 | | < .001 | | 0.834 | |
| NBC_T3 | | 0.756 | | 0.041 | | < .001 | | 0.645 | |
| NBD_T4 | | 1.192 | | 0.039 | | < .001 | | 0.841 | |
| NBL_T4 | | 1.051 | | 0.037 | | < .001 | | 0.871 | |
| NBC_T4 | | 0.756 | | 0.041 | | < .001 | | 0.696 | |
| RML_T1 | | 0.927 | | 0.062 | | < .001 | | 0.319 | |
| RMC_T1 | | 1.073 | | 0.062 | | < .001 | | 0.333 | |
| RML_T2 | | 0.927 | | 0.062 | | < .001 | | 0.419 | |
| RMC_T2 | | 1.073 | | 0.062 | | < .001 | | 0.517 | |
| RML_T3 | | 0.927 | | 0.062 | | < .001 | | 0.577 | |
| RMC_T3 | | 1.073 | | 0.062 | | < .001 | | 0.569 | |
| RML_T4 | | 0.927 | | 0.062 | | < .001 | | 0.602 | |
| RMC_T4 | | 1.073 | | 0.062 | | < .001 | | 0.646 | |
| FSL_T1 | | 1.013 | | 0.07 | | < .001 | | 0.413 | |
| FSC_T1 | | 0.987 | | 0.07 | | < .001 | | 0.396 | |
| FSL_T2 | | 1.013 | | 0.07 | | < .001 | | 0.513 | |
| FSC_T2 | | 0.987 | | 0.07 | | < .001 | | 0.649 | |
| FSL_T3 | | 1.013 | | 0.07 | | < .001 | | 0.596 | |
| FSC_T3 | | 0.987 | | 0.07 | | < .001 | | 0.504 | |
| FSL_T4 | | 1.013 | | 0.07 | | < .001 | | 0.54 | |
| FSC_T4 | | 0.987 | | 0.07 | | < .001 | | 0.465 | |
| SUD_T1 | | 0.897 | | 0.045 | | < .001 | | 0.568 | |
| SUC_T1 | | 1.103 | | 0.045 | | < .001 | | 0.691 | |
| SUD_T2 | | 0.897 | | 0.045 | | < .001 | | 0.545 | |
| SUC_T2 | | 1.103 | | 0.045 | | < .001 | | 0.647 | |
| SUD_T3 | | 0.897 | | 0.045 | | < .001 | | 0.739 | |
| SUC_T3 | | 1.103 | | 0.045 | | < .001 | | 0.806 | |
| SUD_T4 | | 0.897 | | 0.045 | | < .001 | | 0.716 | |
| SUC_T4 | | 1.103 | | 0.045 | | < .001 | | 0.787 | |
| Note: Parameter estimates reflect the Strong invariant model. T=task phase. | | | | | | | | | |
