# Supplementary material for: From task-general towards task-specific cognitive operations in a few minutes? Working memory performance as an adaptive process
Source: Q J Exp Psychol (Hove). 2024 Sep 18;78(8):1547–63. doi: 10.1177/17470218241278272 (PMC12267864; doi:10.1177/17470218241278272)
Supplement: sj-docx-3-qjp-10.1177_17470218241278272 – Supplemental material for From task-general towards task-specific cognitive operations in a few minutes? Working memory performance as an adaptive process [file sj-docx-3-qjp-10.1177_17470218241278272.docx]

Appendix C: Unconstrained Correlation Matrix

| C1: Experiment 1 Unconstrained Correlation Matrix | | | | | | | | | | | | |
| --- | --- | --- | --- | --- | --- | --- | --- | --- | --- | --- | --- | --- |
|  | Correlations | | | | | | | | | | | |
|  |  | | | | | | | | | | | |
|  | NB T1 | RM T1 | SP T1 | NB T2 | RM T2 | SP T2 | NB T3 | RM T3 | SP T3 | NB T4 | RM T4 | SP T4 |
| NB T1 | 1.000 |  |  |  |  |  |  |  |  |  |  |  |
| RM T1 | 0.987 | 1.000 |  |  |  |  |  |  |  |  |  |  |
| SP T1 | 0.999 | 0.939 | 1.000 |  |  |  |  |  |  |  |  |  |
| NB T2 | 1.003 | 0.839 | 0.839 | 1.000 |  |  |  |  |  |  |  |  |
| RM T2 | 0.992 | 1.121 | 1.193 | 0.891 | 1.000 |  |  |  |  |  |  |  |
| SP T2 | 0.806 | 0.844 | 1.150 | 0.715 | 1.131 | 1.000 |  |  |  |  |  |  |
| NB T3 | 0.993 | 0.733 | 0.748 | 1.011 | 0.924 | 0.670 | 1.000 |  |  |  |  |  |
| RM T3 | 0.846 | 1.202 | 0.904 | 0.773 | 1.229 | 0.855 | 0.791 | 1.000 |  |  |  |  |
| SP T3 | 0.825 | 0.899 | 1.130 | 0.728 | 1.243 | 0.995 | 0.677 | 0.901 | 1.000 |  |  |  |
| NB T4 | 0.983 | 0.719 | 0.725 | 0.996 | 0.796 | 0.656 | 0.994 | 0.724 | 0.662 | 1.000 |  |  |
| RM T4 | 0.790 | 0.921 | 0.872 | 0.666 | 1.287 | 0.971 | 0.650 | 0.908 | 0.790 | 0.624 | 1.000 |  |
| SP T4 | 0.918 | 0.976 | 1.110 | 0.769 | 1.068 | 1.024 | 0.684 | 0.898 | 1.039 | 0.670 | 0.766 | 1.000 |

Note. NB=N-back, RM=Running Memory, SP=Span. T=Task phase.

| C2: Experiment 2 Unconstrained Correlation Matrix | | | | | | | | | | | | | | | | |
| --- | --- | --- | --- | --- | --- | --- | --- | --- | --- | --- | --- | --- | --- | --- | --- | --- |
|  | Correlations | | | | | | | | | | | | | | | |
|  | NB T1 | RM T1 | SP T1 | SU T1 | NB T2 | RM T2 | SP T2 | SU T2 | NB T3 | RM T3 | SP T3 | SU T3 | NB T4 | RM T4 | SP T4 | SU T4 |
| NB T1 | 1.000 |  |  |  |  |  |  |  |  |  |  |  |  |  |  |  |
| RM T1 | 0.266 | 1.000 |  |  |  |  |  |  |  |  |  |  |  |  |  |  |
| SP T1 | 0.428 | 1.309 | 1.000 |  |  |  |  |  |  |  |  |  |  |  |  |  |
| SU T1 | 0.480 | 0.994 | 0.870 | 1.000 |  |  |  |  |  |  |  |  |  |  |  |  |
| NB T2 | 1.086 | 0.249 | 0.428 | 0.620 | 1.000 |  |  |  |  |  |  |  |  |  |  |  |
| RM T2 | 0.306 | 1.555 | 0.881 | 0.832 | 0.315 | 1.000 |  |  |  |  |  |  |  |  |  |  |
| SP T2 | 0.321 | 0.793 | 0.914 | 0.524 | 0.342 | 0.455 | 1.000 |  |  |  |  |  |  |  |  |  |
| SU T2 | 0.497 | 0.976 | 0.901 | 1.056 | 0.616 | 0.828 | 0.428 | 1.000 |  |  |  |  |  |  |  |  |
| NB T3 | 1.125 | 0.252 | 0.323 | 0.478 | 0.989 | 0.274 | 0.311 | 0.514 | 1.000 |  |  |  |  |  |  |  |
| RM T3 | 0.496 | 1.154 | 0.906 | 0.803 | 0.542 | 1.222 | 0.569 | 0.813 | 0.479 | 1.000 |  |  |  |  |  |  |
| SP T3 | 0.447 | 1.135 | 0.896 | 0.727 | 0.532 | 0.758 | 0.594 | 0.596 | 0.451 | 0.784 | 1.000 |  |  |  |  |  |
| SU T3 | 0.473 | 0.778 | 0.817 | 1.026 | 0.545 | 0.738 | 0.416 | 1.139 | 0.477 | 0.778 | 0.594 | 1.000 |  |  |  |  |
| NB T4 | 1.158 | 0.237 | 0.395 | 0.466 | 0.985 | 0.267 | 0.331 | 0.529 | 1.006 | 0.486 | 0.430 | 0.479 | 1.000 |  |  |  |
| RM T4 | 0.441 | 1.108 | 0.622 | 0.801 | 0.450 | 1.003 | 0.457 | 0.718 | 0.458 | 0.813 | 0.452 | 0.686 | 0.444 | 1.000 |  |  |
| SP T4 | 0.465 | 1.333 | 0.956 | 0.958 | 0.554 | 1.105 | 0.980 | 0.925 | 0.452 | 0.939 | 1.068 | 0.804 | 0.457 | 0.765 | 1.000 |  |
| SU T4 | 0.495 | 0.778 | 0.662 | 1.017 | 0.538 | 0.831 | 0.368 | 1.056 | 0.507 | 0.787 | 0.601 | 1.026 | 0.509 | 0.639 | 0.702 | 1.000 |
|  |  |  |  |  |  |  |  |  |  |  |  |  |  |  |  |  |

Note. NB=N-back, RM=Running Memory, SP=Span, SU=Selective Updating, T=task phase.
