# Supplementary material for: From task-general towards task-specific cognitive operations in a few minutes? Working memory performance as an adaptive process
Source: Q J Exp Psychol (Hove). 2024 Sep 18;78(8):1547–63. doi: 10.1177/17470218241278272 (PMC12267864; doi:10.1177/17470218241278272)
Supplement: sj-docx-4-qjp-10.1177_17470218241278272 – Supplemental material for From task-general towards task-specific cognitive operations in a few minutes? Working memory performance as an adaptive process [file sj-docx-4-qjp-10.1177_17470218241278272.docx]

| **Table D1.** Descriptive statistics of performances in each task phase in Experiment 2 | | | | | | | |
| --- | --- | --- | --- | --- | --- | --- | --- |
|  | **N** | **M** | **SD** | **Skewness** | **SE of Skewness** | **Kurtosis** | **SE of Kurtosis** |
| **Task phase 1** | | | | | | | |
| FSC | 196 | 0.604 | 0.329 | -0.118 | 0.174 | -1.327 | 0.346 |
| FSL | 201 | 0.7 | 0.326 | -0.67 | 0.172 | -0.885 | 0.341 |
| NBC | 196 | 1.898 | 0.529 | 0.63 | 0.174 | 0.288 | 0.346 |
| NBD | 199 | 1.637 | 0.34 | -0.277 | 0.172 | -1.278 | 0.343 |
| NBL | 199 | 1.98 | 0.599 | 0.592 | 0.172 | -0.309 | 0.343 |
| RMC | 196 | 2.571 | 1.553 | -0.496 | 0.174 | -1.34 | 0.346 |
| RML | 200 | 2.76 | 1.419 | -0.688 | 0.172 | -0.977 | 0.342 |
| SUC | 196 | 3.008 | 1.214 | -0.275 | 0.174 | -0.631 | 0.346 |
| SUD | 201 | 3.032 | 1.267 | -0.155 | 0.172 | -0.695 | 0.341 |
| **Task phase 2** | | | | | | | |
| FSC | 196 | 0.575 | 0.269 | -0.196 | 0.174 | -0.719 | 0.346 |
| FSL | 201 | 0.689 | 0.345 | -0.7 | 0.172 | -0.888 | 0.341 |
| NBC | 196 | 2.435 | 0.725 | 0.174 | 0.174 | -0.847 | 0.346 |
| NBD | 199 | 2.645 | 0.913 | 0.605 | 0.172 | 0.023 | 0.343 |
| NBL | 201 | 2.39 | 0.744 | 0.145 | 0.172 | -0.611 | 0.341 |
| RMC | 196 | 2.742 | 1.072 | -0.628 | 0.174 | -0.457 | 0.346 |
| RML | 200 | 2.815 | 1.089 | -0.689 | 0.172 | -0.285 | 0.342 |
| SUC | 196 | 3.033 | 1.222 | -0.346 | 0.174 | -0.447 | 0.346 |
| SUD | 201 | 3.032 | 1.188 | -0.069 | 0.172 | -0.753 | 0.341 |
| **Task phase 3** | | | | | | | |
| FSC | 196 | 0.567 | 0.266 | -0.267 | 0.174 | -0.648 | 0.346 |
| FSL | 201 | 0.693 | 0.236 | -0.58 | 0.172 | -0.223 | 0.341 |
| NBC | 196 | 2.861 | 1.009 | 0.705 | 0.174 | 0.657 | 0.346 |
| NBD | 199 | 3.007 | 1.38 | 1.146 | 0.172 | 1.036 | 0.343 |
| NBL | 201 | 2.763 | 1.105 | 0.922 | 0.172 | 0.644 | 0.341 |
| RMC | 196 | 2.676 | 1.135 | -0.552 | 0.174 | -0.602 | 0.346 |
| RML | 200 | 2.868 | 0.991 | -0.61 | 0.172 | -0.547 | 0.342 |
| SUC | 196 | 3.083 | 1.193 | -0.359 | 0.174 | -0.624 | 0.346 |
| SUD | 201 | 3.008 | 1.096 | -0.172 | 0.172 | -0.636 | 0.341 |
| **Task phase 4** | | | | | | | |
| FSC | 196 | 0.552 | 0.291 | -0.215 | 0.174 | -0.983 | 0.346 |
| FSL | 201 | 0.699 | 0.255 | -0.763 | 0.172 | -0.085 | 0.341 |
| NBC | 195 | 2.879 | 1.111 | 0.653 | 0.174 | 0.148 | 0.346 |
| NBD | 199 | 3.193 | 1.542 | 1.181 | 0.172 | 0.93 | 0.343 |
| NBL | 201 | 2.864 | 1.33 | 0.978 | 0.172 | 0.476 | 0.341 |
| RMC | 196 | 2.686 | 1.136 | -0.49 | 0.174 | -0.796 | 0.346 |
| RML | 200 | 2.822 | 1.066 | -0.698 | 0.172 | -0.314 | 0.342 |
| SUC | 196 | 3.068 | 1.251 | -0.294 | 0.174 | -0.864 | 0.346 |
| SUD | 201 | 3.103 | 1.074 | -0.16 | 0.172 | -0.189 | 0.341 |
| FSC = Forward span with colors; FSL = Forward span with letters; NBC = n-back with colors; NBD = N-back with digits; NBL = N-back with letters; RMC= Running memory with colors; RML = Running memory with letters; SUC = Selective updating with colors; SUD = Selective updating with digits. | | | | | | | |

**Table D2.** Intercorrelations between all the variables across the four task phases in Experiment 2. The number after the abbreviation (e.g., FSC1) indicates the task phase. FSC = Forward span with colors; FSL = Forward span with letters; NBC = n-back with colors; NBD = N-back with digits; NBL = N-back with letters; RMC= Running memory with colors; RML = Running memory with letters; SUC = Selective updating with colors; SUD = Selective updating with digits. (Note: please copy and paste to another program such as Excel to ease viewing. Data used in the analysis is available at <https://osf.io/gvqhu/>)

| **Pearson's Correlations** | | | | | | | | | | | | | | | | | | | | | | | | | | | | | | | | | | | | | |
| --- | --- | --- | --- | --- | --- | --- | --- | --- | --- | --- | --- | --- | --- | --- | --- | --- | --- | --- | --- | --- | --- | --- | --- | --- | --- | --- | --- | --- | --- | --- | --- | --- | --- | --- | --- | --- | --- |
| **Variable** |  | **FSC1** | **FSC2** | **FSC3** | **FSC4** | **FSL1** | **FSL2** | **FSL3** | **FSL4** | **NBC1** | **NBC2** | **NBC3** | **NBC4** | **NBD1** | **NBD2** | **NBD3** | **NBD4** | **NBL1** | **NBL2** | **NBL3** | **NBL4** | **RMC1** | **RMC2** | **RMC3** | **RMC4** | **RML1** | **RML2** | **RML3** | **RML4** | **SUC1** | **SUC2** | **SUC3** | **SUC4** | **SUD1** | **SUD2** | **SUD3** | **SUD4** |
| 1. FSC1 | Pearson's r | — |  |  |  |  |  |  |  |  |  |  |  |  |  |  |  |  |  |  |  |  |  |  |  |  |  |  |  |  |  |  |  |  |  |  |  |
| 2. FSC2 | Pearson's r | 0.19 | — |  |  |  |  |  |  |  |  |  |  |  |  |  |  |  |  |  |  |  |  |  |  |  |  |  |  |  |  |  |  |  |  |  |  |
| 3. FSC3 | Pearson's r | 0.205 | 0.324 | — |  |  |  |  |  |  |  |  |  |  |  |  |  |  |  |  |  |  |  |  |  |  |  |  |  |  |  |  |  |  |  |  |  |
| 4. FSC4 | Pearson's r | 0.109 | 0.341 | 0.399 | — |  |  |  |  |  |  |  |  |  |  |  |  |  |  |  |  |  |  |  |  |  |  |  |  |  |  |  |  |  |  |  |  |
| 5. FSL1 | Pearson's r | 0.204 | 0.233 | 0.183 | 0.14 | — |  |  |  |  |  |  |  |  |  |  |  |  |  |  |  |  |  |  |  |  |  |  |  |  |  |  |  |  |  |  |  |
| 6. FSL2 | Pearson's r | 0.158 | 0.326 | 0.082 | 0.246 | 0.162 | — |  |  |  |  |  |  |  |  |  |  |  |  |  |  |  |  |  |  |  |  |  |  |  |  |  |  |  |  |  |  |
| 7. FSL3 | Pearson's r | 0.2 | 0.277 | 0.296 | 0.317 | 0.245 | 0.261 | — |  |  |  |  |  |  |  |  |  |  |  |  |  |  |  |  |  |  |  |  |  |  |  |  |  |  |  |  |  |
| 8. FSL4 | Pearson's r | 0.245 | 0.332 | 0.277 | 0.22 | 0.804 | 0.226 | 0.363 | — |  |  |  |  |  |  |  |  |  |  |  |  |  |  |  |  |  |  |  |  |  |  |  |  |  |  |  |  |
| 9. NBC1 | Pearson's r | 0.15 | 0.169 | 0.156 | 0.189 | 0.182 | 0.205 | 0.298 | 0.254 | — |  |  |  |  |  |  |  |  |  |  |  |  |  |  |  |  |  |  |  |  |  |  |  |  |  |  |  |
| 10. NBC2 | Pearson's r | 0.208 | 0.138 | 0.157 | 0.171 | 0.18 | 0.205 | 0.273 | 0.256 | 0.563 | — |  |  |  |  |  |  |  |  |  |  |  |  |  |  |  |  |  |  |  |  |  |  |  |  |  |  |
| 11. NBC3 | Pearson's r | 0.158 | 0.236 | 0.191 | 0.215 | 0.19 | 0.213 | 0.208 | 0.231 | 0.645 | 0.678 | — |  |  |  |  |  |  |  |  |  |  |  |  |  |  |  |  |  |  |  |  |  |  |  |  |  |
| 12. NBC4 | Pearson's r | 0.112 | 0.221 | 0.117 | 0.08 | 0.244 | 0.158 | 0.239 | 0.241 | 0.653 | 0.622 | 0.794 | — |  |  |  |  |  |  |  |  |  |  |  |  |  |  |  |  |  |  |  |  |  |  |  |  |
| 13. NBD1 | Pearson's r | 0.108 | 0.047 | 0.03 | 0.033 | 0.034 | 0.117 | 0.195 | 0.119 | 0.182 | 0.184 | 0.219 | 0.149 | — |  |  |  |  |  |  |  |  |  |  |  |  |  |  |  |  |  |  |  |  |  |  |  |
| 14. NBD2 | Pearson's r | 0.088 | 0.091 | 0.09 | 0.131 | 0.079 | 0.096 | 0.333 | 0.206 | 0.566 | 0.394 | 0.413 | 0.43 | 0.502 | — |  |  |  |  |  |  |  |  |  |  |  |  |  |  |  |  |  |  |  |  |  |  |
| 15. NBD3 | Pearson's r | 0.027 | 0.097 | 0.125 | 0.148 | 0.053 | 0.086 | 0.222 | 0.198 | 0.635 | 0.455 | 0.528 | 0.567 | 0.299 | 0.763 | — |  |  |  |  |  |  |  |  |  |  |  |  |  |  |  |  |  |  |  |  |  |
| 16. NBD4 | Pearson's r | 0.106 | 0.13 | 0.122 | 0.138 | 0.075 | 0.129 | 0.234 | 0.206 | 0.693 | 0.476 | 0.555 | 0.603 | 0.255 | 0.714 | 0.944 | — |  |  |  |  |  |  |  |  |  |  |  |  |  |  |  |  |  |  |  |  |
| 17. NBL1 | Pearson's r | 0.195 | 0.114 | 0.103 | 0.104 | 0.087 | 0.153 | 0.224 | 0.17 | 0.623 | 0.454 | 0.531 | 0.574 | 0.275 | 0.648 | 0.804 | 0.866 | — |  |  |  |  |  |  |  |  |  |  |  |  |  |  |  |  |  |  |  |
| 18. NBL2 | Pearson's r | 0.177 | 0.172 | 0.099 | 0.101 | 0.066 | 0.149 | 0.18 | 0.155 | 0.534 | 0.388 | 0.542 | 0.597 | 0.222 | 0.457 | 0.462 | 0.497 | 0.543 | — |  |  |  |  |  |  |  |  |  |  |  |  |  |  |  |  |  |  |
| 19. NBL3 | Pearson's r | 0.177 | 0.171 | 0.15 | 0.074 | 0.124 | 0.161 | 0.274 | 0.216 | 0.727 | 0.441 | 0.584 | 0.661 | 0.223 | 0.574 | 0.621 | 0.663 | 0.657 | 0.732 | — |  |  |  |  |  |  |  |  |  |  |  |  |  |  |  |  |  |
| 20. NBL4 | Pearson's r | 0.178 | 0.216 | 0.147 | 0.126 | 0.192 | 0.092 | 0.263 | 0.273 | 0.817 | 0.453 | 0.593 | 0.658 | 0.216 | 0.556 | 0.654 | 0.691 | 0.677 | 0.634 | 0.847 | — |  |  |  |  |  |  |  |  |  |  |  |  |  |  |  |  |
| 21. RMC1 | Pearson's r | 0.142 | 0.215 | 0.193 | 0.247 | 0.165 | 0.169 | 0.248 | 0.223 | 0.105 | 0.17 | 0.111 | 0.086 | 0.095 | 0.156 | 0.121 | 0.109 | 0.143 | 0.109 | 0.066 | 0.128 | — |  |  |  |  |  |  |  |  |  |  |  |  |  |  |  |
| 22. RMC2 | Pearson's r | 0.197 | 0.138 | 0.208 | 0.255 | 0.169 | -0.004 | 0.136 | 0.297 | 0.191 | 0.158 | 0.118 | 0.089 | -0.035 | 0.073 | 0.12 | 0.091 | 0.024 | 0.021 | 0.069 | 0.119 | 0.251 | — |  |  |  |  |  |  |  |  |  |  |  |  |  |  |
| 23. RMC3 | Pearson's r | 0.235 | 0.289 | 0.266 | 0.277 | 0.15 | 0.135 | 0.231 | 0.246 | 0.212 | 0.225 | 0.202 | 0.181 | 0.122 | 0.209 | 0.237 | 0.244 | 0.229 | 0.179 | 0.246 | 0.28 | 0.261 | 0.449 | — |  |  |  |  |  |  |  |  |  |  |  |  |  |
| 24. RMC4 | Pearson's r | 0.268 | 0.18 | 0.187 | 0.193 | 0.116 | 0.178 | 0.159 | 0.24 | 0.293 | 0.258 | 0.263 | 0.232 | 0.049 | 0.165 | 0.22 | 0.223 | 0.182 | 0.207 | 0.267 | 0.291 | 0.252 | 0.437 | 0.54 | — |  |  |  |  |  |  |  |  |  |  |  |  |
| 25. RML1 | Pearson's r | 0.219 | 0.124 | 0.149 | 0.201 | 0.134 | 0.097 | 0.234 | 0.19 | 0.09 | 0.014 | 0.016 | -0.034 | -0.024 | -0.012 | 0.054 | 0.033 | -0.029 | -0.093 | 0.008 | 0.078 | 0.102 | 0.26 | 0.181 | 0.223 | — |  |  |  |  |  |  |  |  |  |  |  |
| 26. RML2 | Pearson's r | 0.142 | 0.237 | 0.153 | 0.201 | 0.178 | 0.136 | 0.308 | 0.273 | 0.208 | 0.16 | 0.038 | 0.105 | -0.049 | 0.105 | 0.154 | 0.161 | 0.131 | 0.1 | 0.128 | 0.182 | 0.199 | 0.197 | 0.24 | 0.249 | 0.262 | — |  |  |  |  |  |  |  |  |  |  |
| 27. RML3 | Pearson's r | 0.194 | 0.18 | 0.243 | 0.203 | 0.289 | 0.12 | 0.247 | 0.35 | 0.301 | 0.235 | 0.211 | 0.208 | 0.124 | 0.188 | 0.183 | 0.179 | 0.166 | 0.217 | 0.197 | 0.27 | 0.251 | 0.401 | 0.335 | 0.345 | 0.267 | 0.315 | — |  |  |  |  |  |  |  |  |  |
| 28. RML4 | Pearson's r | 0.087 | 0.195 | 0.148 | 0.238 | 0.171 | 0.132 | 0.126 | 0.259 | 0.282 | 0.225 | 0.234 | 0.265 | -0.047 | 0.136 | 0.235 | 0.24 | 0.211 | 0.169 | 0.2 | 0.229 | 0.226 | 0.312 | 0.231 | 0.385 | 0.161 | 0.307 | 0.445 | — |  |  |  |  |  |  |  |  |
| 29. SUC1 | Pearson's r | 0.242 | 0.26 | 0.235 | 0.325 | 0.214 | 0.147 | 0.327 | 0.296 | 0.313 | 0.291 | 0.319 | 0.279 | 0.116 | 0.254 | 0.179 | 0.199 | 0.214 | 0.332 | 0.294 | 0.285 | 0.196 | 0.313 | 0.284 | 0.325 | 0.195 | 0.235 | 0.364 | 0.327 | — |  |  |  |  |  |  |  |
| 30. SUC2 | Pearson's r | 0.245 | 0.217 | 0.19 | 0.255 | 0.284 | 0.069 | 0.218 | 0.362 | 0.275 | 0.229 | 0.27 | 0.257 | 0.092 | 0.256 | 0.238 | 0.267 | 0.227 | 0.267 | 0.232 | 0.303 | 0.247 | 0.327 | 0.29 | 0.323 | 0.126 | 0.159 | 0.261 | 0.169 | 0.591 | — |  |  |  |  |  |  |
| 31. SUC3 | Pearson's r | 0.21 | 0.266 | 0.271 | 0.313 | 0.346 | 0.139 | 0.247 | 0.398 | 0.32 | 0.259 | 0.327 | 0.346 | 0.196 | 0.286 | 0.243 | 0.254 | 0.248 | 0.316 | 0.321 | 0.334 | 0.212 | 0.348 | 0.336 | 0.325 | 0.198 | 0.181 | 0.38 | 0.332 | 0.621 | 0.679 | — |  |  |  |  |  |
| 32. SUC4 | Pearson's r | 0.143 | 0.268 | 0.248 | 0.302 | 0.219 | 0.095 | 0.268 | 0.284 | 0.386 | 0.321 | 0.369 | 0.408 | 0.107 | 0.279 | 0.325 | 0.327 | 0.265 | 0.323 | 0.354 | 0.359 | 0.233 | 0.358 | 0.323 | 0.262 | 0.111 | 0.295 | 0.358 | 0.33 | 0.592 | 0.659 | 0.72 | — |  |  |  |  |
| 33. SUD1 | Pearson's r | 0.173 | 0.204 | 0.235 | 0.347 | 0.191 | 0.124 | 0.211 | 0.292 | 0.304 | 0.271 | 0.269 | 0.224 | 0.218 | 0.264 | 0.229 | 0.226 | 0.189 | 0.212 | 0.251 | 0.301 | 0.234 | 0.263 | 0.26 | 0.326 | 0.213 | 0.12 | 0.254 | 0.306 | 0.401 | 0.406 | 0.509 | 0.481 | — |  |  |  |
| 34. SUD2 | Pearson's r | 0.13 | 0.201 | 0.183 | 0.251 | 0.202 | 0.061 | 0.18 | 0.265 | 0.289 | 0.267 | 0.31 | 0.266 | 0.208 | 0.251 | 0.25 | 0.239 | 0.206 | 0.238 | 0.252 | 0.285 | 0.213 | 0.285 | 0.278 | 0.288 | 0.171 | 0.085 | 0.311 | 0.315 | 0.4 | 0.358 | 0.509 | 0.474 | 0.594 | — |  |  |
| 35. SUD3 | Pearson's r | 0.22 | 0.207 | 0.226 | 0.254 | 0.249 | 0.116 | 0.263 | 0.332 | 0.336 | 0.279 | 0.325 | 0.315 | 0.188 | 0.326 | 0.303 | 0.317 | 0.301 | 0.269 | 0.32 | 0.341 | 0.208 | 0.335 | 0.357 | 0.354 | 0.141 | 0.152 | 0.31 | 0.331 | 0.498 | 0.549 | 0.604 | 0.617 | 0.6 | 0.612 | — |  |
| 36. SUD4 | Pearson's r | 0.262 | 0.136 | 0.243 | 0.22 | 0.176 | 0.081 | 0.229 | 0.264 | 0.31 | 0.222 | 0.347 | 0.297 | 0.248 | 0.287 | 0.278 | 0.303 | 0.29 | 0.237 | 0.232 | 0.291 | 0.291 | 0.342 | 0.32 | 0.331 | 0.133 | 0.072 | 0.35 | 0.299 | 0.483 | 0.469 | 0.573 | 0.554 | 0.567 | 0.58 | 0.606 | — |
|  | | | | | | | | | | | | | | | | | | | | | | | | | | | | | | | | | | | | | |
|  |  |  |  |  |  |  |  |  |  |  |  |  |  |  |  |  |  |  |  |  |  |  |  |  |  |  |  |  |  |  |  |  |  |  |  |  |  |
