# Supplementary material for: From task-general towards task-specific cognitive operations in a few minutes? Working memory performance as an adaptive process
Source: Q J Exp Psychol (Hove). 2024 Sep 18;78(8):1547–63. doi: 10.1177/17470218241278272 (PMC12267864; doi:10.1177/17470218241278272)
Supplement: sj-docx-5-qjp-10.1177_17470218241278272 – Supplemental material for From task-general towards task-specific cognitive operations in a few minutes? Working memory performance as an adaptive process [file sj-docx-5-qjp-10.1177_17470218241278272.docx]

**Appendix E: Analyses without the n-back tasks**

**Experiment 1**

| **Table E1.** Invariance Testing and Model Constraint Evaluation for Experiment 1 | | | | | | | | | | | |
| --- | --- | --- | --- | --- | --- | --- | --- | --- | --- | --- | --- |
|  | *χ^2^* | DF | p-value | CFI | TLI | RMSEA | SRMR | CFI Diff | Δ *χ^2^* | DF Diff | p-value |
| Configural | 235.978 | 192 | 0.017 | 0.970 | 0.957 | 0.028 | 0.047 | NA | NA | NA | NA |
| Weak | 248.814 | 200 | 0.011 | 0.967 | 0.954 | 0.029 | 0.052 | 0.003 | 12.836 | 8 | 0.118 |
| Strong | 267.618 | 212 | 0.006 | 0.962 | 0.951 | 0.030 | 0.054 | 0.005 | 18.804 | 12 | 0.093 |
| Lt.Correlation | 268.762 | 215 | 0.007 | 0.964 | 0.953 | 0.029 | 0.054 | NA | 1.144 | 3 | 0.766 |
| Lt.Variance | 274.326 | 218 | 0.006 | 0.962 | 0.952 | 0.030 | 0.056 | NA | 6.708 | 6 | 0.349 |
| Omnibus | 274.705 | 220 | 0.007 | 0.963 | 0.954 | 0.029 | 0.056 | NA | 7.087 | 8 | 0.527 |

| **Table E2.** Covariances and Standard Deviations for Experiment 1 | | | | | | | | |
| --- | --- | --- | --- | --- | --- | --- | --- | --- |
|  | RM 1 | SP 1 | RM 2 | SP 2 | RM 3 | SP 3 | RM 4 | SP 4 |
| RM 1 | 1.000 |  |  |  |  |  |  |  |
| SP 1 | 0.898 | 1.000 |  |  |  |  |  |  |
| RM 2 | 1.158 | 0.958 | 1.000 |  |  |  |  |  |
| SP 2 | 0.891 | 1.024 | 1.000*† | 1.000 |  |  |  |  |
| RM 3 | 1.388 | 0.825 | 1.205 | 0.884 | 1.000 |  |  |  |
| SP 3 | 1.001 | 1.01 | 1.156 | 1.005 | 1.000*† | 1.000 |  |  |
| RM 4 | 0.995 | 0.796 | 1.244 | 1.000 | 0.945 | 0.837 | 1.000 |  |
| SP 4 | 1.077 | 0.984 | 0.979 | 1.019 | 0.978 | 1.078 | 0.806 | 1.000 |
|  |  |  |  |  |  |  |  |  |
|  | RM 0 | SP 0 | RM 1 | SP 1 | RM 2 | SP 2 | RM 3 | SP 3 |
| SD | 0.494† | 0.133† | 0.494† | 0.133† | 0.494† | 0.133† | 0.494† | 0.133† |
| Note: An asterisk (*) indicates a parameter that has been constrained to be equivalent. RM=Running memory, SP=Span. The number  after the paradigm acronym refers to the task phase. | | | | | | | | |

**Experiment 2**

| **Table E3.** Invariance Testing and Model Constraint Evaluation for Experiment 2 | | | | | | | | | | | |
| --- | --- | --- | --- | --- | --- | --- | --- | --- | --- | --- | --- |
|  | *χ^2^* | DF | *p*-value | CFI | TLI | RMSEA | SRMR | CFI Diff | Δ *χ^2^* | DF Diff | *p*-value |
| Configural | 149.275 | 162 | 0.755 | 1 | 1.013 | 0 | 0.043 | NA | NA | NA | NA |
| Weak | 143.923 | 159 | 0.798 | 1 | 1.015 | 0 | 0.037 | 0 | 5.352 | 3 | 0.148 |
| Strong | 149.290 | 168 | 0.847 | 1 | 1.018 | 0 | 0.038 | 0 | 5.367 | 9 | 0.801 |
| Lt.Correlation | 154.439 | 177 | 0.888 | 1 | 1.021 | 0 | 0.039 | NA | 5.149 | 9 | 0.821 |
| Lt.Variance | 154.560 | 175 | 0.865 | 1 | 1.019 | 0 | 0.041 | NA | 5.27 | 7 | 0.627 |
| Omnibus | 162.675 | 183 | 0.858 | 1 | 1.018 | 0 | 0.046 | NA | 13.385 | 15 | 0.573 |

| **Table E4.** Covariances and Standard Deviations for Experiment 2 | | | | | | | | | | | | |
| --- | --- | --- | --- | --- | --- | --- | --- | --- | --- | --- | --- | --- |
|  | RM 1 | SP 1 | SU 1 | RM 2 | SP 2 | SU 2 | RM 3 | SP 3 | SU 3 | RM 4 | SP 4 | SU 4 |
| RM 1 | 1.000 |  |  |  |  |  |  |  |  |  |  |  |
| SP 1 | 1.000* | 1.000 |  |  |  |  |  |  |  |  |  |  |
| SU 1 | 1.000*† | 1.000* | 1.000 |  |  |  |  |  |  |  |  |  |
| RM 2 | 1.301 | 0.833 | 0.933 | 1.000 |  |  |  |  |  |  |  |  |
| SP 2 | 0.657 | 0.890 | 0.569 | 0.417 | 1.000 |  |  |  |  |  |  |  |
| SU 2 | 0.978 | 0.974 | 1.118 | 1.000*† | 0.449 | 1.000 |  |  |  |  |  |  |
| RM 3 | 1.021 | 0.873 | 0.880 | 1.165 | 0.555 | 0.896 | 1.000 |  |  |  |  |  |
| SP 3 | 0.967 | 0.896 | 0.809 | 0.719 | 0.595 | 0.651 | 0.780*† | 1.000 |  |  |  |  |
| SU 3 | 0.716 | 0.844 | 1.084 | 0.785 | 0.414 | 1.165 | 0.780*† | 0.609 | 1.000 |  |  |  |
| RM 4 | 1.060 | 0.622 | 0.975 | 1.015 | 0.460 | 0.887 | 0.824 | 0.472 | 0.784 | 1.000 |  |  |
| SP 4 | 1.112 | 0.956 | 1.095 | 1.058 | 0.977 | 1.027 | 0.931 | 1.079 | 0.846 | 0.780*† | 1.000 |  |
| SU 4 | 0.764 | 0.719 | 1.082 | 0.893 | 0.381 | 1.091 | 0.829 | 0.636 | 1.032 | 0.780*† | 0.780* | 1.000 |
|  |  |  |  |  |  |  |  |  |  |  |  |  |
|  | RM 0 | SP 0 | SU 0 | RM 1 | SP 1 | SU 1 | RM 2 | SP 2 | SU 2 | RM 3 | SP 3 | SU 3 |
| SD | 0.586† | 0.137 | 0.697† | 0.586† | 0.173 | 0.697† | 0.586† | 0.137† | 0.870† | 0.586† | 0.137† | 0.870† |
| Note: An asterisk (*) indicates a parameter that has been constrained to be equivalent. RM=Running Memory, SP=Span, SU=Selective Updating. The number after the paradigm acronym refers to the task phase. | | | | | | | | | | | | |
